# G6PD Deficiency and Antimalarial Efficacy for Uncomplicated Malaria in Bangladesh: A Prospective Observational Study

**DOI:** 10.1371/journal.pone.0154015

**Published:** 2016-04-29

**Authors:** Benedikt Ley, Mohammad Shafiul Alam, Kamala Thriemer, Mohammad Sharif Hossain, Mohammad Golam Kibria, Sarah Auburn, Eugenie Poirot, Ric N. Price, Wasif Ali Khan

**Affiliations:** 1 Global and Tropical Health Division, Menzies School of Health Research and Charles Darwin University, Darwin, Australia; 2 International Centre for Diarrheal Diseases and Research, Dhaka, Bangladesh; 3 Global Health Group, University of California San Francisco, San Francisco, California, United States of America; 4 Centre for Tropical Medicine and Global Health, Nuffield Department of Clinical Medicine, University of Oxford, Oxford, United Kingdom; The George Washington University School of Medicine and Health Sciences, UNITED STATES

## Abstract

**Background:**

The Bangladeshi national treatment guidelines for uncomplicated malaria follow WHO recommendations but without G6PD testing prior to primaquine administration. A prospective observational study was conducted to assess the efficacy of the current antimalarial policy.

**Methods:**

Patients with uncomplicated malaria, confirmed by microscopy, attending a health care facility in the Chittagong Hill Tracts, Bangladesh, were treated with artemether-lumefantrine (days 0–2) plus single dose primaquine (0.75mg/kg on day2) for *P*. *falciparum* infections, or with chloroquine (days 0–2) plus 14 days primaquine (3.5mg/kg total over 14 days) for *P*. *vivax* infections. Hb was measured on days 0, 2 and 9 in all patients and also on days 16 and 30 in patients with *P*. *vivax* infection. Participants were followed for 30 days. The study was registered with the clinical trials website (NCT02389374).

**Results:**

Between September 2014 and February 2015 a total of 181 patients were enrolled (64% *P*. *falciparum*, 30% *P*. *vivax* and 6% mixed infections). Median parasite clearance times were 22.0 (Interquartile Range, IQR: 15.2–27.3) hours for *P*. *falciparum*, 20.0 (IQR: 9.5–22.7) hours for *P*. *vivax* and 16.6 (IQR: 10.0–46.0) hours for mixed infections. All participants were afebrile within 48 hours, two patients with *P*. *falciparum* infection remained parasitemic at 48 hours. No patient had recurrent parasitaemia within 30 days. Adjusted male median G6PD activity was 7.82U/gHb. One male participant (1/174) had severe G6PD deficiency (<10% activity), five participants (5/174) had mild G6PD deficiency (10–60% activity). The Hb nadir occurred on day 2 prior to primaquine treatment in *P*. *falciparum* and *P*. *vivax* infected patients; mean fractional fall in Hb was -8.8% (95%CI -6.7% to -11.0%) and -7.4% (95%CI: -4.5 to -10.4%) respectively.

**Conclusion:**

The current antimalarial policy remains effective. The prevalence of G6PD deficiency was low. Main contribution to haemolysis in G6PD normal individuals was attributable to acute malaria rather than primaquine administration.

**Trial Registration:**

ClinicalTrials.gov NCT02389374

## Introduction

In Bangladesh approximately 14 million people are at risk of malaria infection, with 13 out of 64 districts reporting clinical cases in 2012 [1]. While there has been a steady decrease in malaria over the last decade [1], this decline has been modest in the Chittagong Hill Tracts (CHT) in the east of the country, which continues to be most heavily affected by malaria [2, 3]. National treatment guidelines for the treatment of uncomplicated malaria follow WHO recommendations [4] with artemether–lumefantrine (AL) plus a single dose of primaquine (SDPQ) recommended for patients with *Plasmodium falciparum* infection, and chloroquine (CQ) plus a 14 day regimen of primaquine (14DPQ) for those with *P*. *vivax* infection. G6PD testing is not done on a routine basis before primaquine treatment. Patients co-infected with *P*. *falciparum* and *P*. *vivax* are treated with AL plus 14DPQ.

Artemisinin resistant *P*. *falciparum* has been reported from Cambodia [5–10], Vietnam [11, 9] and lately from the Thai-Myanmar border [12, 13, 9]. The CHT are located on the Bangladesh- Myanmar border and are a potential gateway for the spread of artemisinin resistance from the Mekong region into the Indian subcontinent and onwards into Africa. The last systematic evaluation of the national guidelines for the treatment of uncomplicated malaria was conducted in 2009 [14], with a subsequent trial of artesunate monotherapy in 2012 [15]. Both studies demonstrated the high efficacy of artemisinin against *P*. *falciparum* in this region. The WHO world malaria report 2015 reports failure rates following AL treatment ranging from 0% to 11.1% on day 28 in Bangladesh in patients with *P*. *falciparum* infection [16].

Drug resistant *P*. *vivax*, has been slower to emerge than *P*. *falciparum*, although there is evidence that the efficacy of CQ is declining across much of the vivax endemic world [17, 18, 16]. The efficacy of CQ against *P*. *vivax* in Bangladesh remains unknown.

Primaquine is the only drug currently available with hypnozoitocidal activity against *P*. *vivax* and gametocytocidal activity against *P*. *falciparum* [19]. Whilst it is well tolerated in the majority of recipients, PQ can cause severe haemolysis in glucose-6-phosphate dehydrogenase (G6PD) deficient individuals [20]. A recent surveillance in the CHT reported prevalence rates of severe and mild G6PD deficiency of 6.4% and 35.0% respectively among the indigenous population [21]. In Bangladesh G6PD deficiency is not tested routinely prior to PQ administration. Although no formal reporting system exists to record drug induced haemolytic events, anecdotal evidence from local clinicians suggests that 14DPQ is well tolerated and that severe drug induced haemolysis is uncommon.

The aim of this study was to assess the efficacy of the recommended schizontocidal regimens and to measure the prevalence of G6PD deficiency and haematological consequences among non-severe G6PD deficient patients with malaria in the CHT of Bangladesh.

## Materials and Methods

### Study site

The study was carried out at the Alikadam Upazilla Health Complex (UHC) in the district of Bandarban, in the Chittagong Hill Tracts (CHT) of Bangladesh (Latitude 21.652380N, Longitude 92.311957E). The area is hilly and mostly forested, with a peak malaria season between May and October. Key mosquito vectors in this region include *Anopheles vagus*, *An*. *nivipes*, *An*. *maculatus*, *An*. *jeyporiensis* and *An*. *barbirostris* [22, 23]. The population is of mixed ethnicity with indigenous as well as Bengali people living in the area. In contrast to the rest of the country, the population density is considerably lower [24].

### Study design and sample size

The study was designed as an observational cohort study with 28 days follow up, based on WHO recommendations [25], and modified to include mixed infections and any level of parasitaemia. To calculate sample size, treatment failure rates of 7% in *P*. *falciparum* to AL and 4% in *P*. *vivax* to CQ were assumed [16]. 101 patients with *P*. *falciparum* infection and 60 patients with *P*. *vivax* infection had to be enrolled to achieve results with 95% confidence and a precision around the estimate of 5%. Assuming a 20% lost to follow up rate during the 30 day follow up period the total sample size was 120 patients with *P*. *falciparum* infection and 72 patients with *P*. *vivax* infection [25].

### Patients

Patients with slide-confirmed malaria (*P*. *falciparum*, *P*. *vivax* or mixed infections) and fever or a history of fever during the preceding 48 hours who presented to the Alikadam UHC were screened for eligibility. Pregnant or lactating women and children under 1 year of age or less than 6 kg in weight were excluded, as were those with danger signs or signs of severity [26], parasitaemia >4%, haemoglobin concentration below 8g/dL, severe malnutrition, known hypersensitivity to any of the study drugs or a comorbidity requiring hospitalisation. Severe malnutrition was defined as a child with growth standard below –3 z-score, symmetrical oedema involving at least the feet or mid-upper arm circumference less than 110 mm. Patients were enrolled irrespective of their G6PD status, but were subsequently excluded prior to primaquine treatment if diagnosed with severe G6PD deficiency. In the absence of a population specific G6PD cut-off activity a cut-off activity of 1.2 U/gHb was applied, an enzyme activity within the range of the fluorescent spot tests cut-off activity [19, 27].

### Study Procedures

Eligible patients were offered to participate and once written informed consent had been granted, they were enrolled and treated according to the national treatment guidelines. A standardized data collection form, modified from the “Primaquine Roll Out Monitoring Pharmacovigilance Tool” developed by the University of California, San Francisco, USA, and adapted for all PQ use, was administered during patient visits [28]. At enrolment demographic data, symptom details and history of antimalarial medication within the previous 14 days was collected. A clinical examination was carried out and venous or capillary blood taken for blood film examination and measurement of haemoglobin concentration and G6PD enzyme activity. Patients were examined twice daily for the first three days and then on day 7, 14, 21 and 28 days after initiation of PQ treatment (corresponding to day 9, 16, 23 and 30 after enrolment). At each follow up visit, a full physical examination was performed, a symptom questionnaire completed, adverse events assessed and blood taken for repeat blood film examination. Haemoglobin (Hb) concentration (HemoCue™ Hb201+, Angelholm, Sweden) was measured on days 0, 2 and 9 in patients with *P*. *falciparum* and on every follow up visit for all other participants. In patients receiving 14DPQ a pill count was conducted on day 16 at the end of PQ treatment and Meth–hemoglobin (MethHb) measured (Masimo™, Masimo Corporation, USA) [29]. Capillary blood was collected on filter paper (Whatman 3MM chromatography paper) on day 0 and the day of failure (S1 Fig).

### Laboratory procedures

Thick and thin films were stained with Giemsa and counted per 200 white blood cells or 2000 red blood cells. All slides were read by two independent readers and the mean of both readings was recorded. Whenever discordant results were obtained, slides were re-read at a reference laboratory (International Centre for Diarrheal Diseases and Research, Bangladesh (icddr,b), Dhaka) and the expert reading was considered as final.

Venous blood samples were stored at 4–8°C and transferred to the reference laboratory within 48 hours for quantitative G6PD measurement. Spectrophotometry was conducted using kits and controls from Randox (UK) on a Shimadzu 1800™ (Japan) spectrophotometer. Enzyme activity was calculated per Hb concentration using the Hb value recorded at the time of sample collection.

### Treatment

When treatment was directly observed, medication was provided with a banana and fatty sweets. All drug doses were administered according to the national antimalarial treatment guidelines. Patients were provided with schizontocidal treatment on days 0, 1 and 2 and if the G6PD measurement from spectrophotometry indicated a G6PD activity above 1.2 U/gHb patients were provided with PQ on day 2.

Patients with *P*. *falciparum* mono- infection were treated with AL (20mg of artemether and 120mg of lumefantrine; Arexel, Jayson Pharmaceuticals, Bangladesh) twice daily for three days (at enrolment and at 8, 24, 36, 48, and 60 hours) according to the patient’s weight: 5–14 kg received one tablet per dose; 15–24 kg, two tablets per dose; 25–34 kg three tablets per dose and >34 kg four tablets per dose. Schizontocidal treatment was followed by a single dose of PQ (15mg per tablet; Kanaprim, Globe Pharmaceuticals, Bangladesh) according to the patients’ weight: 5–9 kg received 0.5 tablets, 10–19 kg received 1 tablet, 20–29 kg received 1.5 tablets, 30–39kg received 2 tablets, 40–49kg tablets received 2.5 tablets and patients 50 kg and more received 3 tablets.

Patients with *P*. *vivax* mono-infections were treated with CQ (Jasochlor, Jayson Pharmaceuticals, Bangladesh), once daily for three days (total dose of 25mg/kg). Schizontocidal treatment was followed on day 2 by a 14 day course of PQ: 6–9 kg, 0.25 tablets / day; 10–19 kg, 0.5 tablets / day; >19kg, 1 tablet / day. Patients with mixed infections received AL plus 14DPQ. Schizontocidal treatment and the first dose of PQ was given supervised all remaining doses of PQ in eligible patients were unsupervised.

Patients with *P*. *falciparum / P*. *vivax* co-infections received the same schizontocidal treatment as those with *P*. *falciparum* mono-infection as well as the radical cure administered to patients with *P*. *vivax* mono-infection.

### Study endpoints

The primary endpoint was the risk of recurrent parasitaemia within 28 days. Secondary endpoints included the proportion of patients with parasitaemia or fever on days 1 and 2 after starting treatment and parasite clearance time and rate [30]. Safety endpoints were the proportion of patients with severe anaemia (Hb<7g/dl) on any day of follow up, the proportion of patients requiring blood transfusion throughout follow up and the proportion of patients with adverse and serious adverse events (AE and SAE). Haematological safety endpoints included the fractional change in Hb between days 0, 2, 9 in patients with *P*. *falciparum* mono-infection and days 0, 2, 9, 16, and 30 in patients with *P*. *vivax* infection either alone or mixed with other species.

### Statistical analysis

Data were double entered and validated using Access software (Microsoft, USA) and analysed using Stata version 14 (Stata Corp., USA). Analysis was based on intention to treat. The Mann-Whitney U test was used for nonparametric comparisons, and Student’s t-test or one-way analysis of variance for parametric comparisons. Proportions were examined using χ^2^ with Yates' correction or Fisher's exact test. Correlations were assessed using the Pearson test for correlated proportions for normal distributed variables and the Spearman rank test for non- normal distributed variables.

Response to treatment was defined according to WHO definitions [25]. Efficacy endpoints were assessed by survival analysis, in which the cumulative risk of failure was calculated by the Kaplan Meier product limit formula [31]. Parasite clearance time (PCT) was defined as the time from drug administration until the first negative malaria slide [32]. The WWARN online parasite clearance estimator (PCE) was used to estimate the parasite clearance rate [33]. Fever clearance time (FCT) in patients with a temperature ≥37.5°C at enrolment, was defined as the time from drug administration until body temperature (axillary) remained below 37.5°C for at least 48 hours.

Recorded G6PD activities were categorized based on the adjusted male median (AMM) [34]. The median of all recorded G6PD activities from male participants was determined and, after excluding samples below the 10^th^ percentile, the revised median was used to define 100% G6PD activity, the AMM. Patients with G6PD activity less than 10% of the AMM were categorised as severely G6PD deficient, those with activity between 10%-60% as mildly G6PD deficient, and those with activity above 60% of the AMM as G6PD normal [35].

The relation of MethHb levels and total dose of PQ received was assessed in all participants receiving 14DPQ in whom a pill count was performed.

### Ethics

The study was approved by the Ethics Review Committee of the icddr,b, Bangladesh and the Human Research Ethics Committee of the Northern Territory Department of Health and Menzies School of Health Research, Australia. Written informed consent was collected from all participants and in case of minors their legal guardians prior to enrolment, in addition written assent was collected from all minors above the age of 11 years. The study was registered with the clinical trials website (http://www.clinicaltrials.gov/ct) as NCT02389374.

## Results

### Baseline

**Fig 1:**
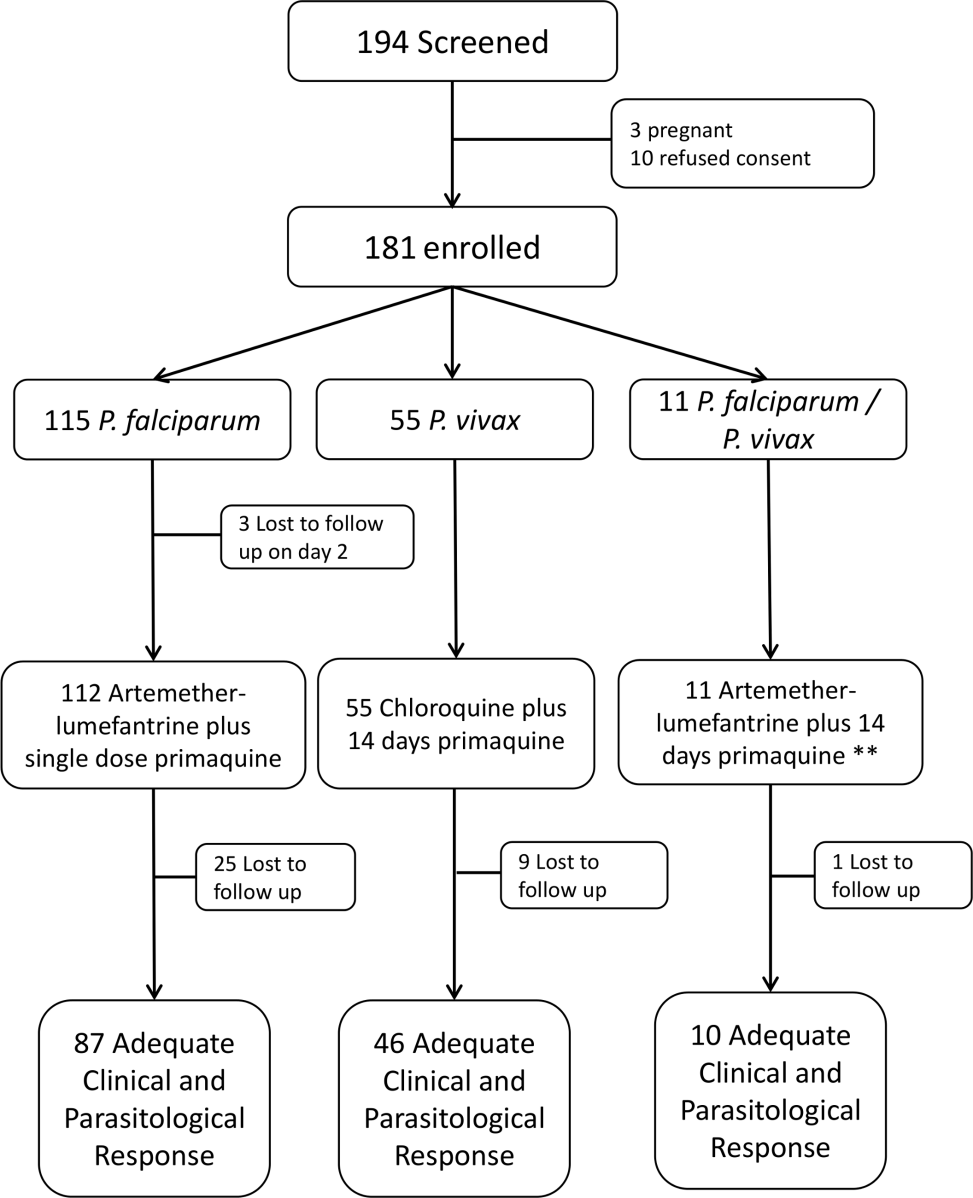
Patient enrolment and clinical outcome. * Full course, ** 14 days primaquine was withheld from one severely deficient participant

Between September 2014 and February 2015, 194 patients presenting to the study hospital were diagnosed with malaria, of whom 181 (93.3%) fulfilled the inclusion criteria and were enrolled. A total of 115 (63.5%) participants had *P*. *falciparum* infection, 55 (30.4%) *P*. *vivax* and 11 (6.1%) mixed infections (Fig 1). Parasite densities were significantly higher in patients with *P*. *falciparum* infection compared to *P*. *vivax* (p = 0.036). The majority of participants (135, 74.6%) were men and this was most apparent in those with *P*. *falciparum* infection (81.7%) compared to *P*. *vivax* (65.5%) infections (OR = 2.36, 95%CI: 1.14 to 4.90, p = 0.021) and compared to mixed (45.5%) infections (OR = 5.37, 95%CI: 1.50 to 19.27, p = 0.011). Overall 66.3% (120/181) of patients had an axillary temperature of more than 37.5°C. A total of 142 (78.5%) patients were of Bengali and 39 (21.5%) of indigenous ethnicity (Table 1). Follow up to the end of the study was achieved in 75.6% (87/115) of patients with *P*. *falciparum* infections, 83.6% (46/55) of those with *P*. *vivax* infection and 90.9% (10/11) of those with mixed infections (Fig 1) with no significant difference between these categories (p = 0.297).

**Table 1:** Baseline characteristics.

	*P*. *falciparum*	*P*. *vivax*	*P*. *falciparum/ P*. *vivax*	*P value* *
**Total**	115	55	11	
**Male** n (%)	94 (81.7%)	36 (65.5%)	5 (45.5%)	0.005
**Median Age in yrs.** (IQR, range)	22 (14–30, 3–66)	18 (11–27, 1–66)	14 (7–26, 6–42)	0.235
**0-5yrs** n (%)	5 (4.3%)	4 (9.0%)	0 (0%)	0.526
**>5-15yrs** n (%)	27 (23.5%)	19 (34.5)	6 (54.5%)	0.049
**>15yrs** n (%)	83 (72.2%)	32 (58.2%)	5 (45.5%)	0.063
**Indigenous ethnicity** n (%)	25 (21.7%)	9 (16.4%)	5 (45.5%)	0.100
**Temp °C** mean (95%CI)	37.8 (37.6 to 38.0)	37.7 (37.4 to 38.0)	38.0 (37.3 to 38.7)	0.629
**Febrile N (%)**	79 (68.7)	34 (61.8)	7 (63.6)	0.662
**Weight (kg)** mean (95%CI)	43.5(40.9–46.1)	39.4 (34.8–44.0)	33.0 (20.8–45.2)	0.041
**Geometric mean parasitaemia (μl^-1^)** (95% CI)	6067 (3897 to 9445)	2704 (1715 to 4264)	10195 (1634 to 63,610)	0.059
**Hb(g/dl) Mean** (95%CI)	13.0 (12.6 to 13.4)	12.0 (11.4–12.5)	12.0 (10.7 to 13.3)	0.007
**Anaemia (<10g/dL)** n (%)	8 (5.5%)	8 (14.9%)	1 (9.1%)	0.284
**Treated with azithromycin, N (%)**	41 (35.7%)	10 (18.2%)	3 (27.3%)	0.065

* overall between species

### G6PD activity

**Fig 2:**
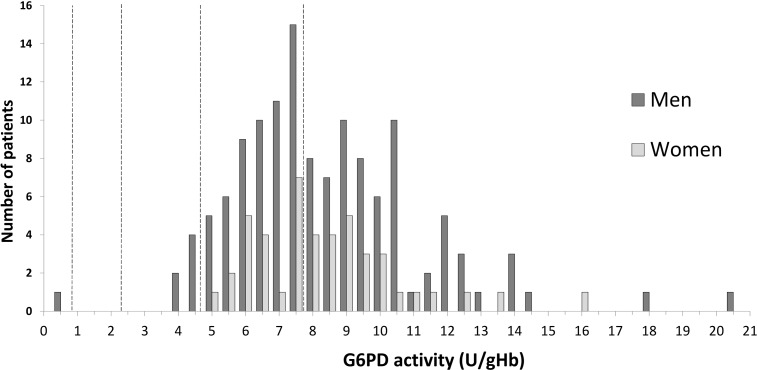
G6PD activity distribution.

G6PD activity was recorded in 175 (96.7%) patients comprising 130 male (74.3%) and 45 female patients (25.7%). The adjusted male median (AMM) of the study population was 7.82 U/gHb. One (0.6%) male participant with a mixed infection was severely deficient (0.70 U/gHb). Mild G6PD deficiency (10–60%) was recorded in five (2.8%) males suffering from *P*. *falciparum* infection but none of the females (Fig 2). All severely and mild G6PD deficient individuals were of Bengali ethnicity.

### *P*. *falciparum* mono-infections

A total of 112 (97.4%) patients completed their schizontocidal treatment, all patients who completed the full course received AL within the target dose range as defined by the WHO [4]. All patients who received schizontocidal treatment also received SDPQ, with mean drug concentrations summarized in Table 2.

**Table 2:** Average total dose of drug (mg/kg) administered. Pf: *Plasmodium falciparum*, Pv: *Plasmodium vivax*, Pf/Pv: mixed infections.

	Infection	n	Mean (95%CI)	Median (IQR)	Range
**Artemether**	Pf	112	10.3 (10.0–10.6)	10.0 (9.2–11.2)	6.8–15.0
**Lumefantrine**	Pf	112	62.0 (60.0–63.9)	60.0 (55.4–67.0)	40.6–90.0
**Primaquine single dose**	Pf	112	0.9 (0.8–0.9)	0.8 (0.8–0.9)	0.6–1.5
**Chloroquine**	Pv	55	27.4 (26.5–28.4)	27.0 (25.0–28.4)	23.1–45.0
**Primaquine radical cure prescribed**	Pv	55	5.2 (4.8–5.7)	4.8 (4.0–6.0)	3.2–10.5
**Primaquine radical cure confirmed by pill count**	Pv	43	5.1 (4.6–5.6)	4.7 (3.7–5.8)	2.8–10.5
**Artemether**	Pf/Pv	11	11.8 (10.0–13.7)	12.0 (8.7–15.0)	8.3–16.0
**Lumefantrine**	Pf/Pv	11	71.1 (59.7–82.4)	72.0 (52.4–90.0)	49.7–96.0
**Primaquine radical cure prescribed**	Pf/Pv	10	6.9 (4.9–8.9)	6.8 (4.6–10.5)	3.6–10.5
**Primaquine radical cure confirmed by pill count**	Pf/Pv	9	6.7 (4.9–8.4)	7.0 (4.9–8.3)	3.6–10.5

No patient vomited any dose of supervised treatment or failed treatment within 30 days. By 24 hours 78.6% (88/112) patients were aparasitemic, with a corresponding median parasite clearance time (PCT) of 22.0 (IQR: 15.2 to 27.3) hours (Fig 2 and Table 3). Two male patients infected with *P*. *falciparum* were still parasitemic at day 2, but were discharged in the absence of clinical symptoms and only seen again on day 6. The first patient had an initial parasite density of 320 parasites μl^-1^, and 200 parasites μl^-1^ 67.3 hours after starting treatment. He had received a total dose of 9.1 mg/kg artemether, 54.3 mg/kg lumefantrine and had received 500mg azithromycin shortly before enrolment. On day 2 the patient received a single dose of 0.85 mg/kg of PQ. The second patient presented with an initial parasitaemia of 19,400 parasites μl^-1^, falling to 1520 μl^-1^ at 59.5 hours after starting 7.5 mg/kg of artemether and 45 mg/kg of lumefantrine, followed by a single dose of PQ (0.7 mg/kg) on day 2. Neither patient had peripheral parasitaemia by microscopy on day six or again during follow up.

**Table 3:** Parasite clearance estimators. PCX: time to clear X% of parasitemia.

	*P*. *falciparum*	*P*. *vivax*	Mixed infections
**Number quantifiable** (%)	**67** (58.3%)	**23** (41.2%)	**4** (36.4%)
*N excluded because of too few data points (%)*	**15** (13.0%)	**16** (29.1%)	**4** (36.4%)
*N excluded because of too low parasitemia (%)*	**28** (24.3%)	**15** (27.3%)	**2** (18.2%)
*N excluded because of other reason (%)*	**5** (4.3%)	**1** (1,8%)	**1** (0.9%)
**Number with lag phase** (%)	**3** (4.5%)	**0**	**0**
**Duration of lag phase in hours** [IQR] (Range)	**10** [9.2–10.0] (8.3–10.0)	**0**	**0**
**Median slope half-life in hours** [IQR] (Range)	**2.8** [2.3–3.4] (1.4–6.3)	**2.7** [2.4–3.2] (2.4–3.2)	**3.3** [2.7–3.8] (1.8–4.5)
**Median PC50 in hours** [IQR] (Range)	**4.4** [3.0–6.0] (0.8–17.8)	**4.01** (3.0–5.3] (0.1–8.3)	**7.9** [6.3–9.4] (1.8–13.6)
**Median PC90 in hours** [IQR] (Range)	**10.9** [8.4–13.9] (4.1–28.4)	**10.8** [8.5–12.1] (5.1–20.4)	**15.5** [12.7–18.2] (6.0–24.1)
**Median PC95 in hours** [IQR] (Range)	**13.7** [10.9–17.5] (5.6–33.0)	**13.7** [10.9–15.3] (7.3–25.6)	**18.8** [15.4–21.9] (7.7–28.6)
**Median PC99 in hours** [IQR] (Range)	**20.1** [16.5–24.8] (8.9–46.3)	**20.2** [16.5–23.1] (12.3–37.7)	**26.5** [21.8–30.7] (11.9–39.0)

A total of 35.7% (41/115) patients received a single dose of azithromycin by the local treating physician. There was no significant difference in the parasite clearance between patients who had and had not received azithromycin at 24 and 48 hours (p = 0.456 and p = 0.654 respectively).

Fever clearance was rapid with 96.6% (106/112) of patients afebrile (<37.5°C) within 24 hours and all patients afebrile by 48 hours. One male and one female were febrile on day 2, however both were aparasitemic at this time and had been afebrile on day of enrolment and day 1. A total of 34.8% (39/112) of patients received a single dose of paracetamol from the local physician before being enrolled by study staff. There was no significant difference in fever clearance in patients who had and had not taken paracetamol (p = 1.000).

A total of six mild to moderate adverse events (AE) were recorded, none of which were related to the treatment. The nadir of the Hb concentration occurred on day 2, with a mean percentage reduction in Hb of 8.8% (95%CI: 6.7% to 11.0%) compared to the day of enrolment (Table 4). Seven (6.3%) patients had a drop in Hb concentration at the nadir greater than 25% (range 25.7% to 33.9%). An additional 7 (6.3%) participants had a fractional fall in Hb ≥ 25% (range -25.4% to -42.1%) between days 0 and 9. In two adult females the Hb concentration fell below 7 g/dL on day 2 (prior to SDPQ administration), one of whom had a Hb of 8.4 g/dL at enrolment falling to 6.2 g/dL, and the other with an Hb of 7.6 g/dL falling to 6.4 g/dL.

**Table 4:** Mean fractional reduction in Hb concentration between baseline and the day of follow up. ND: Not done. Cell values represent the mean % fall (95% CI) and the number of patients (n) included in the analysis.

	*All*	*P*. *falciparum*	*P*. *vivax*	Mixed infections
**Day 2**	**-8.7** (-10.3 to -7.0), n = 176	**-8.8** (-11.0 to -6.7), n = 112	**-7.4** (-10.4 to– 4.5), n = 54	**-13.3** (-19.4 to -7.3), n = 10
**Day 6**	**-6.0** (-8.9 to– 3.2), n = 57	ND	**-5.3** (-8.6 to -2.0), n = 47	-**9.4** (-15.3 to– 3.5), n = 10
**Day 9**	**-6.3** (-8.9 to -3.8), n = 153	**-7.7** (-11.2 to -4.2), n = 95	**-2.3** (-6.2 to +1.5), n = 49	**-13.8** (-22.1 to -5.5), n = 9
**Day 12**	**-2.6** (-6.8 to +1.7), n = 52	ND	**-1.7** (-6.5 to +3.2), n = 44	**-7.5** (-16.1 to +1.1), n = 8
**Day 16**	**-1.8** (-6.1 to +5.1), n = 54	ND	**-0.5** (-6.1 to +5.1), n = 43	**-8.3** (-20.2 to +3.7), n = 9
**Day 23**	**+0.2** (-3.9 to + 5.4), n = 53	ND	**+0.8** (-3.8 to +5.4), n = 45	**-2.7** (-13.4 to +8.0), n = 8
**Day 30**	**-0.3** (-4.1 to + 3.5), n = 56	ND	**+0.2** (-3.7 to + 4.5), n = 46	**-3.3** (-16.8 to + 10.2), n = 9

The Hb on day 2, prior to administering a single dose of PQ, and on day 9 was measured in 95 participants with *P*. *falciparum* infection. The mean fractional difference in Hb between those days was +2.9% (95%CI: -1.14% to +6.8%, n = 91) in G6PD normal patients and -9.1% (95%CI: -53.2% to +35.0%, n = 4) in G6PD deficient patients (p = 0.739).

### *P*. *vivax* mono-infections

All patients (n = 55) completed their schizontocidal treatment and no patient vomited during observed treatment. All 55 patients were treated with 14DPQ with 11 (20.0%) patients prescribed a treatment dose outside the therapeutic dose recommended by the WHO (lower range: 3.2–3.4mg/kg, n = 4; upper range 7.5–10.5, n = 7)[4]. Mean total drug doses are summarized in Table 2.

**Fig 3:**
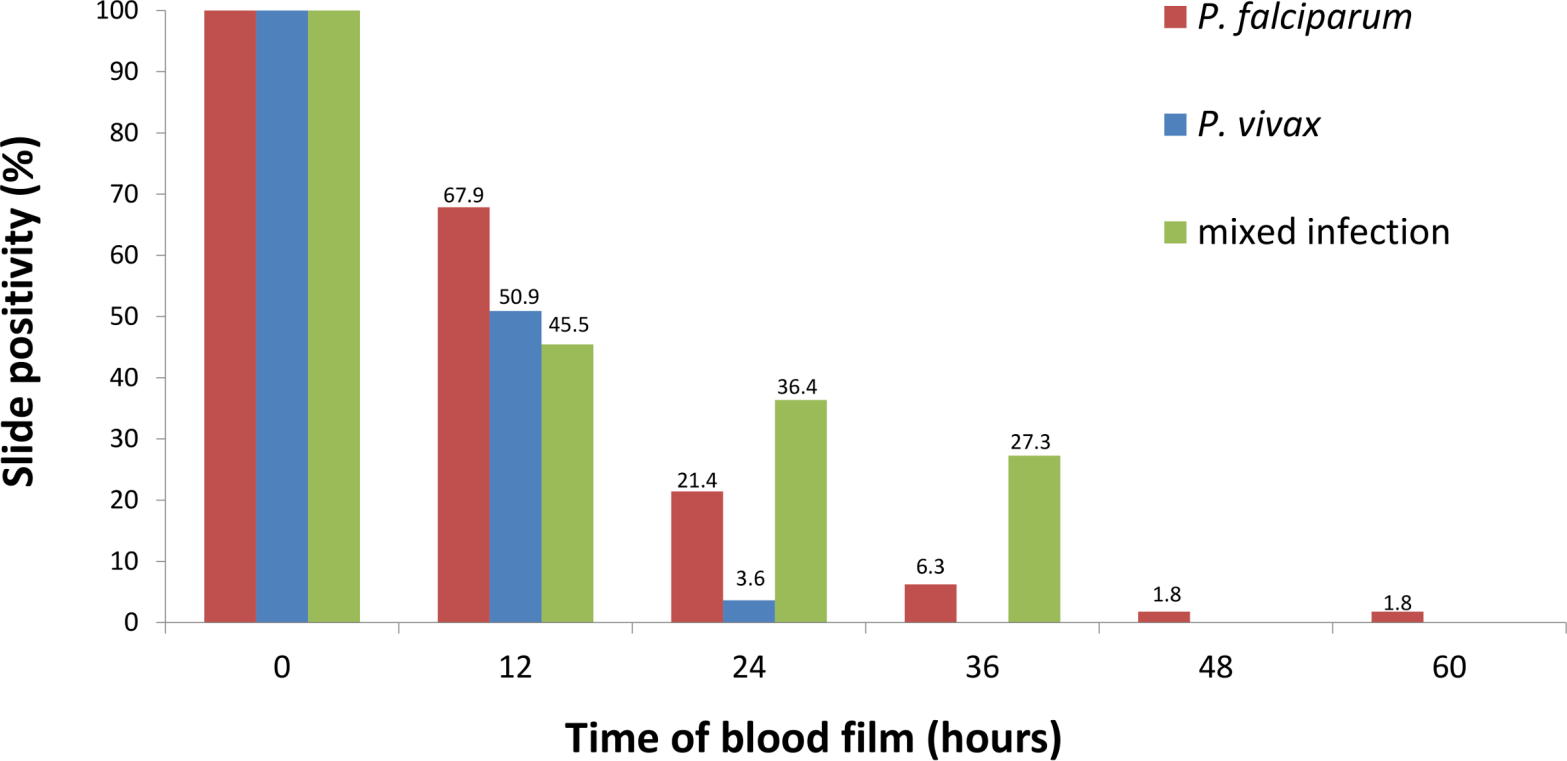
Parasite clearance times. Dotted lines indicate 10%, 30%, 60% and 100% G6PD activity based on the adjusted male median (from left to right).

All but 2 patients (96.4%) were aparasitemic within 24 hours and all patients were aparasitemic within 36 hours (Fig 3). The median parasite clearance time was 20.0 (IQR: 9.5 to 22.7) hours (Table 3). A total of 18.1% (10/55) of patients received a single dose of azithromycin by the local clinician before enrolment. There was no significant difference in parasite clearance at 24 hours between patients with and without antibiotic treatment (p = 0.333).

In total 2 patients (3.6%) remained febrile beyond 24 hours, all of whom had a temperature below 37.5°C 48 hours after start of schizontocidal treatment. 17 patients (30.9%) received a single dose of paracetamol prior to enrolment from the local physician but this had no apparent effect on fever clearance (p = 0.537).

A pill count was conducted in 43 (78.2%) patients, of whom 9 (20.9%) had not completed the full 14 day course of PQ. Three patients missed a single dose (7.0%), four patients missed two doses (9.3%), one patient missed three doses (2.3%) and one patient missed five doses (2.3%) (Table 2).

The Meth-Hb concentration was measured on day 16 at the end of radical cure in all patients (n = 43) who had also undergone a pill count. The mean concentration was 2.05% (95CI: 1.63–2.45%, range 0.10–6.30%), however no significant correlation was found between Meth-Hb concentration and PQ intake based on pill count (r_s_ = -0.193; p = 0.215).

No AEs were recorded in this group. The nadir in Hb concentration was reached on day *5*2 with a mean reduction in Hb of 7.4% (95%CI: 4.5–10.4). One patient had a 28.4% fall in Hb (from 10.2 g/dL on day 0 to 7.3 g/dL on day 2), and another had a 25.5% fall in Hb (from 14.1g/dL on day 2 to 10.5g/dL on day 16 following completion of 14DPQ). By 21 days most patients had returned to their baseline Hb concentration (Table 4).

### *P*. *falciparum / P*. *vivax* co-infections

All patients (n = 11) completed their schizontocidal treatment and no patient vomited their treatment. One patient (9.1%) was excluded from radical cure following measured G6PD activity of 0.7 U/gHb, below the pre-defined threshold value for PQ based radical cure of 1.2 U/gHb,. The mean total dose of drug administered is summarized in Table 2.

By 24 hours 7 patients (63.6%), by 36 hours 8 patients (72.2%) and by 48 hour all patients were aparasitemic. The median parasite clearance time was 16.6 (interquartile range, IQR: 10.0 to 46.0) hours and all patients were afebrile within 24 hours. A pill count performed at the end of PQ treatment on day 16 was performed in 9 (90.0%) patients, of whom 7 had completed the full treatment course and two patients had missed a total of 3 doses each. Meth haemoglobin concentration was measured in the same nine patients, but did not correlate with PQ uptake (r_s_ = 0.053, p = 0.892). The highest value recorded within the entire study population was in a 14 year old male with mixed infection who had received a full course of 14DPQ (total dose 4.9 mg / kg), had normal G6PD activity of 8.53 U/gHb and a Meth-Hb value of 6.7%.

No AEs were recorded in this group. Nadir in Hb was reached on day 9, seven days after start of PQ treatment with a mean drop of 13.8% (95CI: 22.1 to 5.5%) between day 0 and day 9. The biggest drop in Hb was recorded before the start of PQ treatment between day 0 and day 2 with a total drop of 13.3% (95CI: 19.4 to 7.3%).

## Discussion

The current national malaria treatment guidelines in Bangladesh remain effective for both *P*. *vivax* and *P*. *falciparum* malaria, with no documented treatment failures by day 28. Reassuringly, in patients with *P*. *falciparum* the parasite clearance half-life was 2.75 hours with a median PCT of 22.0 hours, values well within the range expected of fully sensitive parasites and similar to earlier reports from this region [14, 32, 36, 37, 9].

Almost half of the samples collected were not included in the WWARN based PCT calculations, hence the speed of parasite clearance may have been overestimated. The parasite clearance half-life, PC50 and PC90 of patients with *P*. *falciparum* infection enrolled in this study were comparable to that observed in studies in other South-East Asian and African areas where parasites remain susceptible to artemisinin, but below that reported from Attapeu, Laos, Ratnakiri, Cambodia where artemisinin resistance is present[9].

Mutations in the kelch 13 gene have been correlated with artemisinin resistance in *P*. *falciparum* [38, 39]. There has been one report from Bangladesh, of a parasite isolate with a mutation of A578S in kelch13, however to date this mutation has not been linked to resistance. [40]. In the current study two patients with *P*. *falciparum* infection were still parasitemic at 48 hours, with minimal decrease in parasite density from baseline. Although blood films were not available at 72 hours, both participants were aparasitemic on day 6 and neither subsequently recurred.

Chloroquine plus primaquine was 100% effective against *P*. *vivax* at day 28, it is however possible that the concomitant administration of 14 days of PQ could have masked low grade CQ resistance [18]. Reassuringly parasite clearance was also rapid with half of the patients clearing their *P*. *vivax* parasitaemia within 12 hours and all by 36 hours. A recent meta–analysis found that clearance of parasites in >95% of participants within 48 hours (as was seen in the current study) is highly indicative of CQ sensitivity [14].

PQ is the only available drug with efficacy against *P*. *vivax* hypnozoites and *P*. *falciparum* late stage gametocytes, however it can cause severe haemolysis in G6PD deficient recipients. The Bangladeshi malaria guidelines recommend the use of single dose PQ for *P*. *falciparum* infection and 14 days for *P*. *vivax* and mixed infections, but prior testing for G6PD deficiency is not routinely done. In a study of indigenous populations in the CHT 6.4% of participants were severely and 35% mildly G6PD deficient [21]. In this study 100% G6PD activity was defined as 11.8 U/ gHb, based on a Cambodian population [41] rather than the adjusted male median of the study population. In the current study 100% G6PD activity was much lower (calculated AMM of 7.82 U/gHb). Whilst this may explain the discrepancy in the fraction of mild G6PDd individuals, the number of severely deficient individuals in this study was also low (0.6%) irrespective of the applied definition of 100% G6PD activity. This may be explained by variation in the ethnicity of the study populations. In the former study almost all patients were from indigenous populations, whereas in the current study almost 80% of participants were of Bengali ethnicity. If true then all or parts of the local indigenous population in the CHT may have higher rates of G6PDd as the general population and therefore may be at greater risk of PQ induced haemolysis than was observed in our study [42, 43].

One patient within the study population was found to have severe G6PD deficiency and was excluded from 14DPQ to prevent drug induced haemolysis. Whilst no serious adverse events (SAEs) were observed in participants receiving a full treatment course, there was a high lost to follow up in patients with *P*. *falciparum* (24%) and *P*. *vivax* mono-infection (16%) and it is possible that some patients could have had an SAE which was not documented by the study staff. However most severe haemolytic events are likely to have occurred within the first 7 days and lost to follow up at this stage only occurred in 7 (6%) patients after SDPQ and 3 (5%) patients after starting primaquine. The lack of other medical facilities within the study area makes it unlikely that patients suffering a haemolytic event would have presented for medical review elsewhere.

Meth-Hb concentration is known to rise during administration of primaquine and was assessed in the study as an indicator for 14DPQ treatment adherence. Although there was no significant correlation between actual dose of primaquine administered and Meth-Haemoglobinaemia the patients smoking habits were not recorded and these may have confounded our observations [44] [45].

Malaria, irrespective of species had a far greater impact on Hb concentrations than the subsequent administration of PQ. While no study participant required a blood transfusion or had apparent haemoglobinuria, one patient (1.5%) with severe G6PD deficiency was excluded from PQ radical cure. Interestingly all other participants suffering from mild G6PD deficiency suffered from a *P*. *falciparum* mono-infection, all participants receiving 14DPQ where classified as G6PD normal. In the remaining patients receiving 14DPQ only one had an Hb drop of greater than 25% (to 10.5gHb/dL) after starting PQ radical cure.

The national treatment guidelines recommend a dose of 0.75mg/kg for SDPQ whereas the current WHO treatment guidelines consider a single dose of 0.25 mg/kg PQ safe even in G6PD deficient patients [4]. This study followed national treatment guidelines, participants received a mean dose of 0.9mg /kg SDPQ and almost 5% of all participants had a fall in Hb greater than 25% within the first seven days after PQ administration.

## Conclusion

The current national malaria treatment guidelines in Bangladesh appear to be efficacious, although the delay in parasite clearance in two patients treated with AL warrants further investigation. The current Bangladeshi recommendations for single dose PQ are higher as suggested by the WHO; reducing the total dose to 0.25 mg/kg should be considered in the absence of routine G6PD testing [46]. The current hypnozoitocidal treatment regimen was safe in this study, although the majority of patients had G6PD activity higher than 60%, and haemolysis may be significantly greater in other indigenous Bangladeshi populations. Further studies to assess the prevalence of G6PD deficiency and haemolytic risk in this region are warranted. In the meantime efforts are required to facilitate G6PD deficiency testing prior to PQ administration.

## Supporting Information

S1 Fig Study Timeline. (DOCX)

S1 File Trial protocol. (DOCX)

S1 Table TREND checklist. (DOCX)
