# Supplementary material for: G6PD Deficiency and Antimalarial Efficacy for Uncomplicated Malaria in Bangladesh: A Prospective Observational Study
Source: PLoS One. 2016 Apr 29;11(4):e0154015. doi: 10.1371/journal.pone.0154015 (PMC4851315; doi:10.1371/journal.pone.0154015)
Supplement: S1 Fig — (DOCX) [file pone.0154015.s001.docx]

**Study timeline**

| **FU Day** | **Day 0** | **Day 1** | **Day 2** | **Day 9** | **Day 16** | **Day 23** | **Day 30** |
| --- | --- | --- | --- | --- | --- | --- | --- |
| **PQ Day** | - | - | 0 | 7 | 14 | 21 | 28 |
| ***Pf* and *Pv* schizontocidal treatment** |  |  |  |  |  |  |  |
| ***Pf* PQ** |  |  |  |  |  |  |  |
| ***Pv* PQ** |  |  |  |  |  |  |  |
| ***Pf* FU days** | X | X | X | X | X | X | X |
| ***Pf* Hb measurement** | X | - | X | X | - | - | - |
| ***Pf* G6PD** | - | - | X | - | - | - | - |
| ***Pv* and *Pf/Pv* FU days** | X | X | X | X | X | X | X |
| ***Pv* and *Pf/Pv* Hb measurement** | X | - | X | X | X | X | X |
| ***Pv* and *Pf/Pv* G6PD** | - | - | X | - | - | - | - |
| ***Pv* and *Pf/Pv* PQ pill count** | - | - | - | - | X | - | - |
| ***Pv* and *Pf/Pv* Meth-Hb measurement** | - | - | - | - | X | - | - |

FU=Follow up; PQ=primaquine treatment; PQ Day= Days after start of PQ; *Pf*= *Plasmodium falciparum; Pv = Plasmodium vivax;* G6PD=spectrophotometry result available; Hb = Hemoglobin; Meth-Hb=Meth-Hemoglobin
