# Supplementary material for: G6PD Deficiency and Antimalarial Efficacy for Uncomplicated Malaria in Bangladesh: A Prospective Observational Study
Source: PLoS One. 2016 Apr 29;11(4):e0154015. doi: 10.1371/journal.pone.0154015 (PMC4851315; doi:10.1371/journal.pone.0154015)
Supplement: S1 File — (DOCX) [file pone.0154015.s002.docx]

**A study to assess primaquine treatment guidelines on malaria in south-east Bangladesh**

**PI: Wasif Khan**

**Co-PIs: Benedikt Ley, Kamala Thriemer, Ric Price, Md Shafiul Alam, Eugenie Poirot, Roly Gosling**

**Version 2.1**

| **SYNOPSIS** |  |
| --- | --- |
| **Title** | **A study to assess primaquine guidelines on malaria treatment in south-east Bangladesh** |
| **Protocol Number** | *--to be added--* |
| **Methodology** | Observational Study |
| **Study Duration** | *P.falciparum* patients to be followed for 30 days  *P.vivax* patients to be followed for 30 days |
| **Country** | Bangladesh |
| **Study Centre** | Alikadam |
| **Primary Objective** | - To assess side effects observed following unsupervised PQ treatment in *P. vivax* and *P. falciparum* patients |
| **Secondary Objectives** | - To assess the schizontocidal efficacy of 3 days Chloroquine (CQ) for *P. vivax* - To assess parasite clearance rates in P*. falciparum* patients treated with artemether-lumefantrine (AL) - To determine the risk of haemolysis associated with G6PD activity - To determine the distribution of G6PD activity and genetic variants among malaria patients - To determine parasite related factors that may affect treatment outcome |
| **Inclusion Criteria** | - Age ≥ 12 months - *P. vivax* or *P. falciparum* monoinfection or *P.v.* / *P.f.* mixed infection - Presence of axillary temperature ≥ 37.5°C or history of fever during the past 24 hrs - Ability to swallow oral medication. - Ability and willingness to comply with the study protocol for the duration of the study - Informed consent/assent from the patient or from a parent or guardian in the case of children. |
| **Exclusion Criteria** | - Presence of general danger signs in children aged under 5 years or signs of severe malaria according to the definitions of WHO - Presence of severe malnutrition - Acute anaemia <8g/dL - Regular medication, which may interfere with antimalarial pharmacokinetics - History of hypersensitivity reactions or contraindications to any of the drug(s) tested or used as alternative treatment(s) - A positive pregnancy test or lactating - Severe G6PD deficient patients will be excluded from PQ treatment |
| **Study design** | This study is a prospective observational cohort study actively monitoring the hematologic response, safety and tolerability of routine treatment of *P. falciparum, P. vivax* and mixed malaria infections (*P. falciparum + P. vivax*). The study will be conducted in Alikadam in the Chittagong Hill Tracts (CHT). |
| **Duration of administration** | - *P. falciparum*: supervised AL for 3 days + single dose PQ - *P. vivax*: Supervised CQ for 3 days + unsupervised PQ for 14 days - Mixed infections: supervised AL for 3 days + unsupervised PQ for 14 days |
| **Duration of patient enrolment** | - Six months |
| **Endpoints** | Safety Endpoints:   - Proportion of patients with severe anaemia (Hb<7g/dl) - Proportion of patients receiving blood transfusion - Fractional change in Hb between baseline and day 9,16, 23 and 30 (incl. proportion of patients with >25% drop in Hb between the timepoints) - Proportion of patients with anaemia less than 8g/dl on day 3 and day 9 - Proportion of patients with adverse and serious adverse events   Efficacy endpoints:   - Proportion of patients with any parasitemia on day 1, 2 and 3 after treatment - Proportion of patients with fever on day 1, 2 and 3 after treatment - Recurrence of parasitaemia within 30 days of follow up   Other Endpoints:   - Proportion of patients adhering to 14 days of primaquine treatment in the vivax cohort as measured by Masimo and pill count - Distribution of G6PD activity among the study population - Frequency and type of variants of the G6PD gene within the study population |
| **Date of first patient enrolled** |  |

Contents

[1. Background 5](#_Toc395861878)

[1.1. Local malaria epidemiology and treatment guidelines 5](#_Toc395861879)

[1.2. Study Site 7](#_Toc395861880)

[**1.3.** **Context of project** 9](#_Toc395861881)

[2. Rationale and Aim 9](#_Toc395861882)

[3. Objective 9](#_Toc395861883)

[4. Methods 10](#_Toc395861884)

[4.1. Study Design 10](#_Toc395861885)

[4.2. Inclusion Criteria 10](#_Toc395861886)

[4.3. Exclusion Criteria 10](#_Toc395861887)

[4.4. Study Procedures 11](#_Toc395861888)

[4.4.1. Treatment & sample collection 11](#_Toc395861889)

[4.4.2. Concomitant treatment 15](#_Toc395861890)

[4.4.3. Rescue treatment 15](#_Toc395861891)

[4.4.4. Loss to follow up 15](#_Toc395861892)

[4.4.5. Patient withdrawal and protocol violation 15](#_Toc395861893)

[4.5. Laboratory and test procedures 16](#_Toc395861894)

[4.5.1. Microscopy 16](#_Toc395861895)

[RDT 16](#_Toc395861896)

[4.5.2. Hemoglobin 16](#_Toc395861897)

[4.5.3. Serology 16](#_Toc395861898)

[4.5.4. Enzyme activity testing 16](#_Toc395861899)

[4.5.5. Methhemoglobin 17](#_Toc395861900)

[4.5.6. Molecular testing (parasite) 17](#_Toc395861901)

[4.5.7. Molecular testing (host) 17](#_Toc395861902)

[5. Assessment of variables and statistical methodology 17](#_Toc395861903)

[5.1. Safety endpoints 17](#_Toc395861904)

[5.2. Efficacy endpoints 17](#_Toc395861905)

[5.3. Other Endpoints 18](#_Toc395861906)

[6. Data Management 18](#_Toc395861907)

[7. Statistical analyses 18](#_Toc395861908)

[8. Sample Size 19](#_Toc395861909)

[9. Ethical considerations 19](#_Toc395861910)

[9.1. Data Safety Monitoring Board (DSMB) 19](#_Toc395861911)

[9.2. Ethical committee (EC) 19](#_Toc395861912)

[9.3. Declaration of Helsinki 20](#_Toc395861913)

[9.4. Expected benefits 20](#_Toc395861914)

[9.5. Potential risks 20](#_Toc395861915)

[9.6. Informed Consent (Annex VIII) 20](#_Toc395861916)

[9.7. Withdrawing informed consent 20](#_Toc395861917)

[9.8. Confidentiality 21](#_Toc395861918)

[9.9. Monitoring and Quality Control 21](#_Toc395861919)

[10. Publication Policy 21](#_Toc395861920)

[11. References 22](#_Toc395861921)

[12. Overview Annexes: 23](#_Toc395861922)

[Annex I 24](#_Toc395861923)

[Annex II 25](#_Toc395861924)

[Annex III 26](#_Toc395861925)

[Annex IV 27](#_Toc395861926)

[Annex V 28](#_Toc395861927)

[Annex VI 29](#_Toc395861928)

[Annex VII 30](#_Toc395861929)

[Annex VIII 31](#_Toc395861930)

[**International Centre for Diarrhoeal Disease Research, Bangladesh** 31](#_Toc395861931)

[**Voluntary Consent Form (for aged 1-10 years)** 31](#_Toc395861932)

[**International Centre for Diarrhoeal Disease Research, Bangladesh** 37](#_Toc395861933)

[**Voluntary Consent Form (for adult)** 37](#_Toc395861934)

[**International Centre for Diarrhoeal Disease Research, Bangladesh** 43](#_Toc395861935)

[**Voluntary Assent Form (for aged 11-17 years)** 43](#_Toc395861936)

[Annex X 72](#_Toc395861937)

[Annex XI 72](#_Toc395861938)

# Background

## Local malaria epidemiology and treatment guidelines

Malaria is endemic in 13 out of 64 districts of Bangladesh, representing a population at risk of about 27 million people. The highest rates of malaria in Bangladesh occur in the Chittagong Hill Districts, and *Plasmodium falciparum* (Pf) is the most prevalent species. Recent data from a cross sectional survey in 2009-2012 found a monthly incidence rate of 0.05/1000 individuals for symptomatic *P. vivax* (Pv) infections and 1.16/1000 for symptomatic *P. falciparum*[[1](#_ENREF_1)]. An earlier survey looking at the overall burden of disease (asymptomatic and symptomatic cases) found a prevalence of 14% for all species combined by microscopy and more than 30% positivity by PCR in Bandarban disctrict [[2](#_ENREF_2)]. In this study *P. falciparum* accounted for 58.9%, *P. vivax* for 13.6%, *P. malariae* for 1.8%, and *P. ovale* for 1.4% of all malaria-positive individuals by PCR. The remaining 24.4% were mixed infections[[2](#_ENREF_2)]. Within the district recent data from the Ministry of Health indicates highest rates in the subdistrict of Alikadam. Malaria is seasonal in the Chittagong Hill Tracks, with the peak transmission between May to October[[2](#_ENREF_2), [3](#_ENREF_3)].

**Figure: Map of Bangladesh with districts coded according to malaria endemicity**


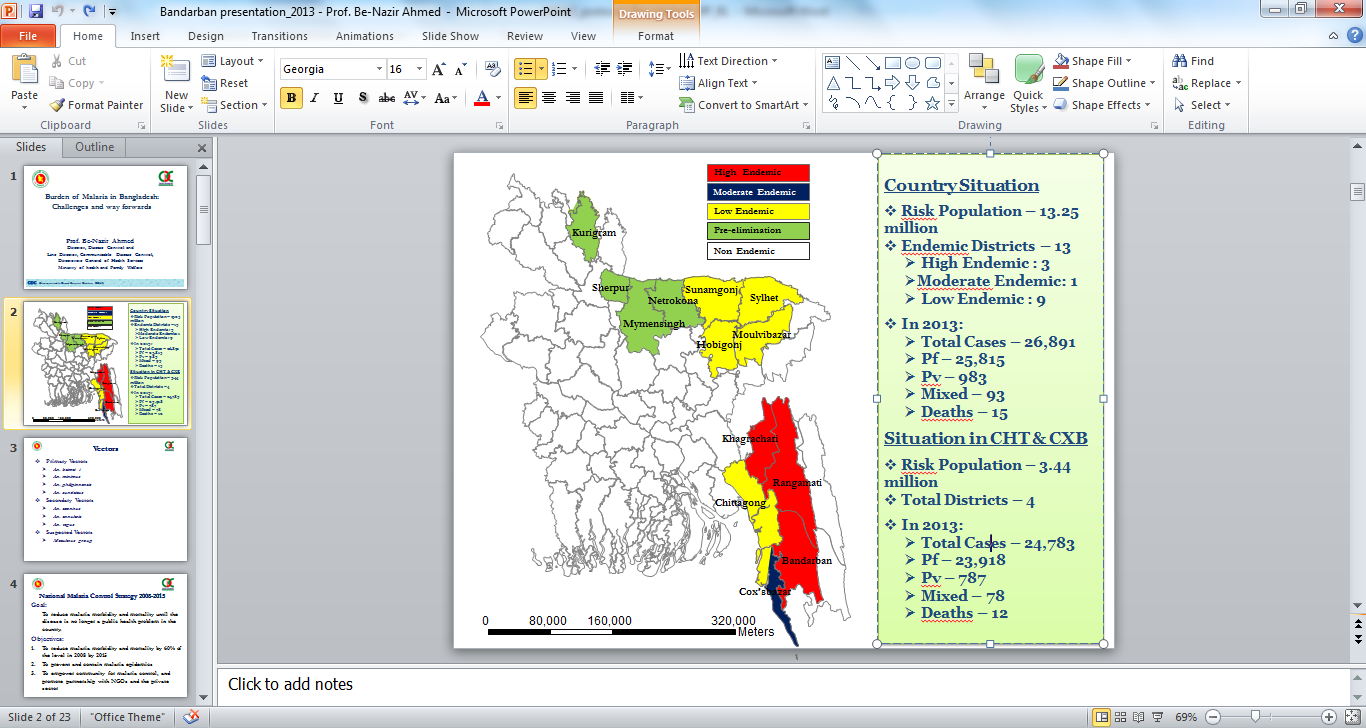


*Source: Prof. Be-Nazir Ahmed, Directorate General of Health Services, Bangladesh Ministry of Health and Family Welfare*

Chloroquine (CQ) has been the primary treatment for blood stages of vivax malaria for almost 60 years, however evidence is gathering for its declining efficacy across much of the vivax endemic world[[4-12](#_ENREF_4)]. Understanding the extent and regional distribution of chloroquine resistance (CQR) in vivax malaria is critical to optimize treatment guidelines, and reduce the risk of recurrent malaria.

For several years artemsinin combination therapy (ACT) is recommended by national guidelines for the treatment of *P.falciparum* infection. However resistance to artesunate has been shown in Cambodia[[13-16](#_ENREF_13)] and more recently along the Thai-Myanmar border[[17](#_ENREF_17)]. In Bangladesh Artemisinin resistance has not been described to date, although the last systematic reports were derived from studies performed in 2009 [9].

Primaquine (PQ) is administered in combination with Chloroquine and ACT without prior testing for Glucose – 6 – Phosphate Dehydrogenase (G6PD) deficiency according to national guidelines. Primaquine is the only drug currently available on the market with hypnozonticidal and, in *P.* f*alciparum* gametocidal, properties[[18](#_ENREF_18)], but can cause severe haemolysis in G6PD deficient individuals, one of the commonest enzyme deficiency in humans, known to be protective against *P. vivax* infections [[19](#_ENREF_19)].

**Primaquine Roll Out Monitoring Pharmacovigilance Tool (PROMPT) program**

This proposed study is part of a multi-center study under the umbrella of the Primaquine Roll Out Monitoring Pharmacovigilance Tool (PROMPT) program. Key objective of this program is to assess and identify operational and clinical obstacles associated with roll out of routine primaquine treatment in malaria. The study is currently being conducted in Swaziland and Senegal. The new site is going to be Bangladesh as well as an additional Asian site to be identified in the coming months

## Study Site

*Alikadam* (Town) has an area of 23.32 sq km. The town has a population of 8326; male 59.44%, female 40.56%. Literacy rate among the town people is 37.1%. The town and district is serviced by one government hospital, the ‘Alikadam Upazilla Health Complex’ with a capacity of 31 beds.

(more details: <http://www.mapsofbangladesh.com/Alikadam-Upazila.php>, last accessed on 02.05.2014)

**Map of Alikadam Subdistrict**

*
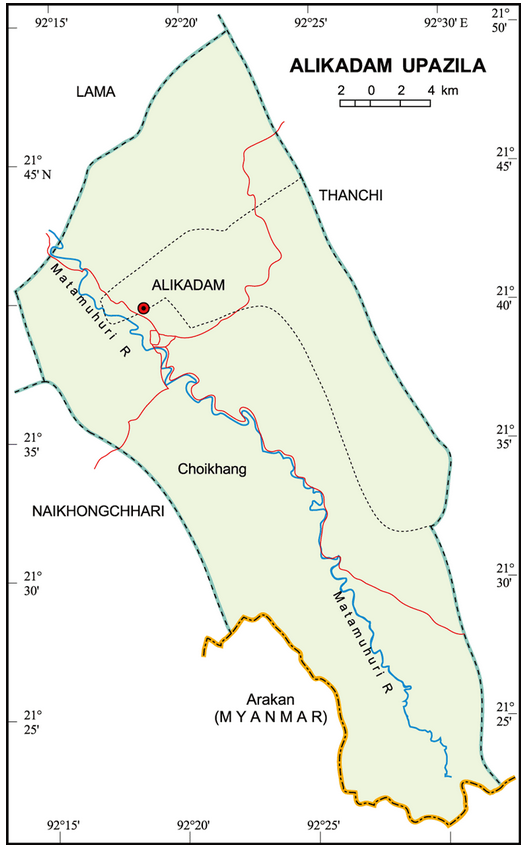
*

Over the last couple of years Alikadam has continuously reported the highest rates of reported and treated malaria infections:

| **Table: Total Malaria 2013 by sub-districts of the Chittagong Hill districts** | | | | |
| --- | --- | --- | --- | --- |
|  | Pf | Pv | MIXED | Grand Total |
| **Bandarban Sadar** | 500 | 11 | 1 | 512 |
| **Alikadam** | 2190 | 142 | 26 | 2358 |
| **Naikongchari** | 1267 | 15 | 13 | 1295 |
| **Ruma** | 678 | 2 | 2 | 682 |
| **Thanchi** | 1084 | 27 | 1 | 1112 |
| **Lama** | 1382 | 82 | 4 | 1468 |
| **Rowangchari** | 1864 | 5 | 0 | 1869 |
| **Sadar Hospital** | 90 | 1 | 0 | 91 |
| **SMO** | 58 | 0 | 0 | 58 |
| Total | 9113 | 285 | 47 | 9445 |

*Source: Ministry of Health and Family Welfare*

In 2013 the hospital has treated a total of 49 P. vivax cases and 432 P. falciparum cases (Ministry of Health and Family Welfare).

- 1. **Context of project**

This project is embedded into the framework of PROMPT (Primaquine Roll Out Monitoring Pharmacovigilance Tool) hosted by the University of California San Francisco (UCSF), USA, and OPRA (OPtimizing the RAdical cure for vivax malaria) hosted by the Menzies School of Health Research (MSHR), Australia, and is facilitated through the International Centre for Diarrheal Diseases, Bangladesh (icddr,b).

## Rationale and Aim

Unpublished data on the prevalence of G6PD deficiency in Bangladesh indicate rates of 20-40% in the local population. Current national guidelines recommend the addition of Primaquine to antimalarial treatment for *P. falciparum* (single dose treatment) and *P. vivax* mono- infections (14days treatment) as well as for *P. falciparum / P. vivax* mixed infections (14 days treatment). In view of lack of available reliable bedside testing primaquine is administered without prior testing of G6PD status. This study aims to assess possible side effects as a result of current national malaria treatment guidelines. To do so we propose an observational study to assess current national treatment guidelines for vivax and falciparum malaria, considering both host and parasite factors that may influence treatment effectiveness.

# Objective

**Primary:**

- To assess side effects observed following unsupervised PQ treatment in *P. vivax* and *P. falciparum* patients

**Secondary:**

- To assess the schizontocidal efficacy of 3 days chloroquine (CQ) for *P. vivax*
- To assess parasite clearance rates in P*. falciparum* patients treated with artemether-lumefantrine (AL)
- To determine the risk of haemolysis associated with G6PD activity
- To determine the distribution of G6PD activity and genetic variants among malaria patients
- To determine parasite related factors that may affect treatment outcome

# Methods

## Study Design

This study is a prospective, observational cohort study actively monitoring the hematologic response, safety and tolerability of routine treatment of *P. falciparum, P. vivax* and mixed malaria infections (*P. falciparum + P. vivax*). The study will be conducted in at a health care facility in Allikadam, in the Chittagong Hill Tracts (CHT), Bangladesh.

## Inclusion Criteria

- Age ≥ 12 months
- *P. vivax* or *P. falciparum* monoinfection or *P.v.* / *P.f.* mixed infection
- Presence of axillary temperature ≥ 37.5°C or history of fever during the past 24 hrs
- Ability to swallow oral medication.
- Ability and willingness to comply with the study protocol for the duration of the study
- Informed consent from the patient or from a parent or guardian in the case of children.

## Exclusion Criteria

- Bodyweight <6kg
- Presence of general danger signs in children aged under 5 years or signs of severe malaria according to the definitions of WHO (Annex III)
- Presence of severe malnutrition^[[1]](#footnote-1)^
- Acute anaemia <8g/dL
- Regular medication, which may interfere with antimalarial pharmacokinetics
- History of hypersensitivity reactions or contraindications to any of the drug(s) tested or used as alternative treatment(s)
- A positive pregnancy test or lactating.
- Severe G6PD deficient patients will be excluded from PQ treatment

## Study Procedures

All patients attending the health care facility will be screened for eligibility, those fulfilling the inclusion and exclusion criteria will be invited to participate in the study.

After provision of written informed consent (Annex VIII) a brief questionnaire will be filled out (Annex IX). Information on demographic background, known hypersensitivity against any of the study drugs and G6PD deficiency will be collected together with the results of a brief physical exam. Depending on body weight, a maximum of 5 ml of venous blood and a fingerprick will be collected from all participants upon enrolment. Additional diagnostic procedures are detailed below.

**Table: Maximum amount of venous blood collected on d0 / body weight**

| **Patient’s approximate weight in kilograms** | **Amount (mL)** |
| --- | --- |
| >6 to 7 | 4-5 |
| >7 | 5 |

All participants will be followed daily until day 2 to provide supervised treatment and ensure clinical and parasitological recovery following chloroquine (Pv arm) or artemether-lumefantrine (Pf arm and Pf/Pv arm) treatment.

Thereafter patients (excluding those with severe G6PD deficiency) will be given unsupervised primaquine for 1 dose (Pf arm) or 14 doses (Pv/mix arm) and reviewed again after 7 days (day 9), 14 days (day 16), 21 days (day 23) and 28 days (day 30). All P. vivax and mixed infections will in addition be followed up after 3 and 10 days (day 6 and 12 respectively) to assure the safety of the provided treatment. Any patient developing signs and symptoms indicative of a malarial episode or an adverse event within the follow up period will be asked to re-attend the clinic for an unscheduled visit, where they will be offered clinical management according to local clinic guidelines. A fingerprick will be performed during every follow up and unscheduled visit, 5ml of blood will be collected for every recurring malaria episode (see table below). Information on adverse events will be collected during every visit. Whenever possible a pill count will be performed on day 16.

### Treatment & sample collection

Patients will receive the following treatment according to national guidelines (Annex II, IV and V) and will be asked to return for follow up:

- **Vivax patients**

CQ (Annex IV) will be administered for three days under direct observation. All patients will be observed for 30 min after drug administration for adverse reactions or vomiting. Any patient who vomits during this observational period will be re-treated with the same dose and observed for an additional 30 min. If the patient vomits the study drug a second time, he or she will receive rescue therapy as defined by the national treatment guidelines (Annex VI).

PQ (Annex V) will be administered unsupervised for 14 days from day 3 – 16.The complete treatment course of PQ will be provided to the patients after completion of schizontocidal treatment. All patients will receive instructions on how to take the drugs and will be asked to return for follow up on, day 6, day 9, day 12, day 16 (at the end of treatment), day 23 and day 30, as well as whenever the patients suffers from signs and symptoms consistent with malaria or an adverse reaction to one of the study drugs. (Annex IX).

- **Falciparum patients:**

AL will be administered twice daily for three days (Annex III). The morning dose will be supervised and the patients given tablets for the evening dose. Whenever supervised treatment is administered, the study patients will be observed for 30 min after drug administration for adverse reactions or vomiting. Any patient who vomits during this observational period will be re-treated with the same dose of medicine and observed for an additional 30 min. If the patient vomits the study drug a second time, he or she will receive rescue therapy (Annex VI). PQ will be given to the patients after schizontocidal treatment is finished and the patients are advised to take the single dose (Annex V) on day 3 unsupervised. All patients will be asked to return for follow up on day 9, day 16, day 23, day 30 and whenever participants suffer from signs and symptoms consistent with malaria or an adverse reaction to one of the study drugs within the follow up period (Annex IX).

- **Patients with mixed infection (Pf and PV):**

AL will be administered twice daily for three days (Annex III). The morning dose will be supervised and the patients given tablets for the evening dose. Whenever supervised treatment is administered, the study patients will be observed for 30 min after drug administration for adverse reactions or vomiting. Any patient who vomits during this observational period will be re-treated with the same dose and observed for an additional 30 min. If the patient vomits the study drug a second time, he or she will receive rescue therapy (Annex VI)

PQ (Annex V) will be administered unsupervised for 14 days from day 3 – 16.The complete treatment course of PQ will be provided to the patients after completion of schizontocidal treatment. All patients will receive instructions on how to take the drugs and will be asked to return for follow up on day 6, day 9, day 12, day 16, day 23, day 30, as well as whenever participants suffer from signs and symptoms consistent with malaria or an adverse reaction to one of the study drugs (Annex IX).

**Table: Treatment, follow up and sample collection schedule *P.f*, *P. vivax* and mixed infections:**

| **Vivax Patients & Patients with mixed infection** | Day 0 | Day 1 | Day 2 | Day 3 | Day 4 | Day 5 | Day 6 | Day 7 | Day 8 | Day 9 | Day 10 | Day 11 | Day 12 | Day 13 | Day 14 | Day 15 | Day 16 | Day 23 | Day 30 | Un-scheduled visit |
| --- | --- | --- | --- | --- | --- | --- | --- | --- | --- | --- | --- | --- | --- | --- | --- | --- | --- | --- | --- | --- |
| FU P.f. patients | X | X | X | (x) |  |  |  |  |  |  |  |  | X |  |  |  | X | X | X |  |
| FU P.v. patients & mixed | X | X | X | (x) |  |  | X |  |  | X |  |  | X |  |  |  | X | X | X |  |
| Consent | X |  | X |  |  |  |  |  |  |  |  |  |  |  |  |  |  |  |  |  |
| Urinary Pregnancy testing (females, 13- 49years) | X |  |  |  |  |  |  |  |  |  |  |  |  |  |  |  |  |  |  |  |
| Capillary Finger Prick | X | X | X |  |  |  |  |  |  | X |  |  |  |  |  |  | X | X | X | X |
| Venous Blood Sample | X |  |  |  |  |  |  |  |  |  |  |  |  |  |  |  |  |  |  | X* |
| Microscopy | X | X | X | (X) |  |  | X |  |  | X |  |  | X |  |  |  | X | X | X | X |
| Hb | X | X | X | (X) |  |  | X |  |  | X |  |  | X |  |  |  | X | X** | X** | X |
| ACT treatment Pf and Pf/Pv | X | X | X |  |  |  |  |  |  |  |  |  |  |  |  |  |  |  |  |  |
| PQ treatment Pf |  |  |  | X |  |  |  |  |  |  |  |  |  |  |  |  |  |  |  |  |
| CQ treatment Pv | X | X | X |  |  |  |  |  |  |  |  |  |  |  |  |  |  |  |  |  |
| PQ treatment Pf and Pf/Pv |  |  |  | X | X | X | X | X | X | X | X | X | X | X | X | X | X |  |  |  |
| Masimo*** |  |  |  |  |  |  |  |  |  |  |  |  |  |  |  |  | X** |  |  | X |
| Pill count |  |  |  |  |  |  |  |  |  |  |  |  |  |  |  |  | X** |  |  |  |
| AE monitoring  *in case of recurrent parasitaemia  ** in Pv and Pf/Pv patients only  ***only in adults and children >2 yearsX | X | X | X |  |  |  |  |  |  | X |  |  |  |  |  |  | X | X | X | X |

## Concomitant treatment

Patients under regular medication at trial entry for conditions other than malaria, will have this documented on the CRF and will be asked to continue medication as usual. Any additional medications taken within the follow-up period must be documented in the CRF. Patients who prematurely discontinue trial medication, or who fail to respond to trial medication and receive other anti-malarial therapy will have this documented. Drugs with antimalarial activity should be avoided (Annex I).

## Rescue treatment

Treatment failure (see Annex VII) are treated according to national guidelines with quinine (Annex VI). This will be noted on the CRF. If the participant has started PQ treatment he will be asked to return for all scheduled follow up visits.

## Loss to follow up

Loss to follow-up occurs when, despite all reasonable efforts, an enrolled patient does not attend for the scheduled visits and cannot be found. Every effort will be made to schedule a follow-up visit for patients who fail to return.

## Patient withdrawal and protocol violation

- Withdrawal of consent. A patient may withdraw consent at any time, without prejudice for further follow-up or treatment at the study site.
- Protocol violation (e.g. enrolment violation or incomplete treatment). Patients with any protocol violation will be followed up as scheduled for safety assessment. This also includes patients with serious adverse events that necessitate termination of treatment before the full course is completed.

# Laboratory and test procedures

## Microscopy

Slides for microscopy will be collected upon enrolment and on all follow up days as well as on any unscheduled visit days.

All slides collected between day 0 and day3 will be re-read by an expert microscopists. Blood smears with discordant results (differences between the two microscopists regarding species diagnosis, parasite density of > 50% or regarding the positivity in general) will be re-examined by a third, independent microscopist, and parasite density will be calculated by averaging the two closest counts.

## RDT

Apart from microscopy all suspected malaria patients will be tested by Rapid Diagnostic Tests (RDT) on day 0. The trade name of the RDT that will be used in this study will be “FalciVax” which is produced by Zephyr Biomedicals, India. Each FalciVax is rapid self-performing, qualitative, two site sandwich immunoassay utilizing whole blood for the detection of *P. falciparum* specific histidine rich protein-2 (Pf, HRP-2) and *P. vivax* specific pLDH. RDT will be performed according to manufacturer instruction (<http://www.tulipgroup.com/Zephyr_New/qrg/falcivax.pdf>.

## Hemoglobin

. Haemoglobin (Hb) will also be measured at baseline and on all follow up visits using a Hemocue Hb (Angelholm, Sweden) machine.

## Serology

Additional serological tests to detect the malaria parasite as well as to assess the response of the host towards the parasite will be performed on collected venous blood at the study laboratory or a reference centre.

## Enzyme activity testing

G6PD activity will be measured using spectrophotometry at the lab maintained by icddr,b for all participants. Samples are transported on a daily basis from the field to the icddr,b lab and results are communicated to the field. A subset of collected samples, tested with spectrophotometry, will be tested with flow cytometry to assess lyonisation at a reference centre capable of performing the respective procedure

## Methhemoglobin

Methemoglobin will be measured by non-invasive methods (Masimo) in vivax patients to determine adherence to unsupervised primaquine treatment

## Molecular testing (parasite)

Parasite DNA will be collected on day 0 and any recurrent parasitaemia. Parasite DNA will be extracted for sequencing and genotyping of the parasite to detect markers of resistance. Molecular testing will be done at the Sanger Institute, UK through Menzies.

## Molecular testing (host)

Based on availability of funds a subset of samples will be tested for known and unknown variants of the G6PD gene. Genotyping and sequencing will be done at the Sanger Insitute, Uk. Host genotyping is strictly related to the G6PD gene (Xq28) and only to achieve the study objectives.

# Assessment of variables and statistical methodology

## Safety endpoints

- Proportion of patients with severe anaemia (Hb<7g/dl)
- Proportion of patients receiving blood transfusion
- Fractional change in Hb between baseline and day 6, 9,12,16, 23 and 30 (incl. proportion of patients with >25% drop in Hb between the timepoints)
- Proportion of patients with anaemia less than 7g/dl on day 3 and day 9
- Proportion of patients with adverse and serious adverse events

## Efficacy endpoints

- Proportion of patients with any parasitemia on day 1, 2 and 3 after treatment
- Proportion of patients with fever on day 1, 2 and 3 after treatment
- Recurrence of parasitaemia within 30 days of follow up
- Proportion of Pf patients with gametocytemia on any of the follow up dates.

## Other Endpoints

- Proportion of patients adhering to 14 days of primaquine treatment in the vivax cohort as measured by Masimo and pill count
- Distribution of G6PD activity among the study population
- Frequency and type of variants of the G6PD gene within the study population

# Data Management

Data will be entered into a custom-made database. Internal error checks and systematic error assessment will be developed to check for data entry discrepancies, invalid data ranges and overall consistency. All discrepancies will be resolved by reference to the original data collection forms.

Original data collection forms will be handled only by staff members and kept under locked storage until completely coded, checked and transported for data entry. Once data entry and cleaning are complete the original forms will be stored as long as required by national laws.

# Statistical analyses

- Safety data will be presented as summary statistics (including number, mean, median, interquartile range, standard deviation, minimum and maximum) of all vital signs variables (values and changes from baseline) will be calculated for each visit by treatment group.
- G6PD activity will be categorised and the relative risk (RR) of a drop of Hb > 25% between day 3 and 9 and day 3 and 16 and day 23 and 30 will be calculated per category for all participants treated with 14 days of PQ.
- Hematological results will be presented as continuous variable of Hb concentration at Day 0, 3, 6, 9, 12 and 16. The fractional fall in Hb between baseline and day 9 and 16, will be calculated, as well as the proportion of patients with anaemia less than 7g/dl between day 3 and day 9.
- Parasite clearance rate will be defined according to the World Wide Antimalarial Resistance Network guidelines (WWARN)[[20](#_ENREF_20)]. The parasite clearance time (PCT) is defined as the time elapsed between the patient’s first dose and the time of the first negative blood slide.
- G6PD variants detected will be presented descriptively, G6PD activity observed will be displayed in the form of a histogram displaying G6PD activity vs. number of patients.

# Sample Size

In 2013 a total of 142 vivax cases and 2190 falciparum cases were registered within the district of Alikadam with the majority of cases occurring during and after the monsoon season. The study hospital treated a total of 49 vivax cases and 432 falciparum cases in the same year. As the proposed study will be conducted during the peak malaria season we propose an enrolment target of approximately 50 vivax cases and 160 falciparum cases.

This study is part of a framework of comparable studies working with similar tools as proposed herein. Results for the primary objective will be pooled together with results of other sites to provide reliable results.

# Ethical considerations

## Data Safety Monitoring Board (DSMB)

A DSMB will be formed at icddr,b to oversee all safty data in regular intervals. The PI in consultation with the IRB Secretariat of icdddr,b will form the DSMB Committee and the frequency of meetings will be agreed on with the Chairman, ERC. It is the principle investigators duty to provide regular reports to the DSMB in due time before the meeting. The DSMB can issue recommendations regarding the further conduct of the study

## Ethical committee (EC)

This study will be submitted for formal review and approval to the Institutional Review Board of the Menzies School of Health Research, the Committee on Human Research (CHR) of the University of San Francisco (UCSF) and the Institutional Ethics Committees at ICDDR,B in Bangladesh. No participant will be enrolled or samples processed before written approval from these bodies is obtained.

## Declaration of Helsinki

The study will be carried out according to the principles stated in the Declaration of Helsinki (Ethical Principles for Medical Research Involving Human Subjects) as amended in 2008, all applicable regulations and according to established international scientific standards.

Any substantial amendments to the protocol or the Informed Consent Form (Annex VIII) will also be submitted for approval to the same ECs and competent authorities and will be implemented only after approval has been obtained.

## Expected benefits

Participants will be provided with a thorough physical examination. The outcome of the re-evaluation of national malaria treatment guidelines and the collection of indicative data on the safety of PQ treatment without prior G6PD testing will be of significant benefit to the local population.

## Potential risks

The study has an observational character: study drugs, treatment regimen and pre-treatment diagnostic tests are identical to national guidelines. Participating in the study does not pose an increased risk compared to standard treatment for the participant.

## Informed Consent (Annex VIII)

The information, consent and assent form will be translated to Bangla and back translated to English to ensure adequate translation. Written informed consent will be obtained from all participants or their legal guardians. Information provided during the consenting process will include description of the sample collection procedure, aim of the study, details on the data collected, potential benefits and risks and assurance of confidentiality for all information and results generated by the study. Legal representatives of enrolled children will be asked for written informed consent. Assent will be sought from all participants ≥11 years and <18 years.

After obtaining the G6PD results a second informed consent process will be conducted to ensure participants understand the potential risks and the expected benefits of Primaquine treatment

Information and consent form will be read out to all participants not fully literate; illiterate patients willing to participate will provide consent by a fingerprint in the presence of a witness.

## Withdrawing informed consent

All participants will be thoroughly informed about their right to withdraw consent at any time without having to provide a reason for withdrawal nor having to fear negative consequences. Data, samples and results collected prior to consent withdrawal will be destroyed.

## Confidentiality

No identifying information will be made public. Identities of all participants are coded with a study identification number (study ID). Only the investigators will have the key that link study ID number to the patient identity.

The study works with a database. Participant data entered into the database is identified through assigned study IDs and the database is password protected. All data analysis is done on the database with the study ID numbers, stored isolates and samples are labelled with the study ID number only. If isolates or samples are shipped to reference laboratories for additional analysis, only study ID number, but not the full name is provided to the receiving organization. Participant’s study information will not be released to anybody outside the medical and research team, except as necessary (and under confidentiality agreement) for the independent monitoring, auditing and for inspection by competent authorities.

## Monitoring and Quality Control

Quality assurance and monitoring will be conducted in regular intervals. Procedural and reagents controls will be provided throughout the study period. Performance of laboratory technicians is assured by on-site training and site visits through the icddr,b and the MSHR. Site-visits are foreseen by the investigators on a regular basis, as well as weekly e-mail or Skype communication with local study staff.

# Publication Policy

All Investigators will be involved in reviewing drafts of the manuscripts, abstracts, press releases and any other publications arising from the study. Authorship will be determined in accordance with the ICMJE guidelines and other contributors will be acknowledged.

# References

1. Ahmed, S., et al., *Malaria hotspots drive hypoendemic transmission in the Chittagong Hill Districts of Bangladesh.* PloS one, 2013. **8**(8): p. e69713.

2. Starzengruber, P., et al., *High prevalence of asymptomatic malaria in south-eastern Bangladesh.* Malar J, 2014. **13**: p. 16.

3. Maude, R.J., et al., *Temporal trends in severe malaria in Chittagong, Bangladesh.* Malar J, 2012. **11**: p. 323.

4. Schwartz, I.K., E.M. Lackritz, and L.C. Patchen, *Chloroquine-resistant Plasmodium vivax from Indonesia.* N Engl J Med, 1991. **324**(13): p. 927.

5. Baird, J.K., et al., *Resistance to chloroquine by Plasmodium vivax in Irian Jaya, Indonesia.* Am J Trop Med Hyg, 1991. **44**(5): p. 547-52.

6. Murphy, G.S., et al., *Vivax malaria resistant to treatment and prophylaxis with chloroquine.* Lancet, 1993. **341**(8837): p. 96-100.

7. Fryauff, D.J., et al., *Chloroquine-resistant Plasmodium vivax in transmigration settlements of West Kalimantan, Indonesia.* Am J Trop Med Hyg, 1998. **59**(4): p. 513-8.

8. Sumawinata, I.W., et al., *Very high risk of therapeutic failure with chloroquine for uncomplicated Plasmodium falciparum and P. vivax malaria in Indonesian Papua.* Am J Trop Med Hyg, 2003. **68**(4): p. 416-20.

9. Ruebush, T.K., 2nd, et al., *Chloroquine-resistant Plasmodium vivax malaria in Peru.* Am J Trop Med Hyg, 2003. **69**(5): p. 548-52.

10. Phan, G.T., et al., *Artemisinin or chloroquine for blood stage Plasmodium vivax malaria in Vietnam.* Trop Med Int Health, 2002. **7**(10): p. 858-64.

11. Kurcer, M.A., Z. Simsek, and Z. Kurcer, *The decreasing efficacy of chloroquine in the treatment of Plasmodium vivax malaria, in Sanliurfa, south-eastern Turkey.* Ann Trop Med Parasitol, 2006. **100**(2): p. 109-13.

12. Teka, H., et al., *Chloroquine-resistant Plasmodium vivax malaria in Debre Zeit, Ethiopia.* Malar J, 2008. **7**: p. 220.

13. Noedl, H., et al., *Evidence of artemisinin-resistant malaria in western Cambodia.* The New England journal of medicine, 2008. **359**(24): p. 2619-20.

14. Noedl, H., et al., *Artemisinin resistance in Cambodia: a clinical trial designed to address an emerging problem in Southeast Asia.* Clinical infectious diseases : an official publication of the Infectious Diseases Society of America, 2010. **51**(11): p. e82-9.

15. Amaratunga, C., et al., *Artemisinin-resistant Plasmodium falciparum in Pursat province, western Cambodia: a parasite clearance rate study.* The Lancet infectious diseases, 2012. **12**(11): p. 851-8.

16. Dondorp, A.M., et al., *Artemisinin resistance in Plasmodium falciparum malaria.* The New England journal of medicine, 2009. **361**(5): p. 455-67.

17. Phyo, A.P., et al., *Emergence of artemisinin-resistant malaria on the western border of Thailand: a longitudinal study.* Lancet, 2012. **379**(9830): p. 1960-6.

18. von Seidlein, L., et al., *Review of key knowledge gaps in glucose-6-phosphate dehydrogenase deficiency detection with regard to the safe clinical deployment of 8-aminoquinoline treatment regimens: a workshop report.* Malar J, 2013. **12**: p. 112.

19. Howes, R.E., et al., *Spatial distribution of G6PD deficiency variants across malaria-endemic regions.* Malar J, 2013. **12**: p. 418.

20. WWARN. *Parasite Clearance Estimator*. 2012 April 2013]; Available from: <http://www.wwarn.org/research/parasite-clearance-estimator>.

# Overview Annexes:

- Annex I: Drugs with antimalarial activity
- Annex II: Severe malaria
- Annex III: Dosing for AL
- Annex IV: Dosing for CQ
- Annex V: Dosing for PQ
- Annex VI: Dosing of 2^nd^ line treatment and treatment for severe malaria
- Annex VII: Definition of treatment failures.
- Annex VIII: Consent Forms
- Annex IX: Case Record Forms
- Annex X: Signs and Symptom for unscheduled visit
- Annex XI: timeline

# Annex I

**Drugs with antimalarial activity that should not be used during the study**

- Proguanil, Chlorproguanil, Pyrimethamine
- Sulfadoxine, Sulfalene, Sulfamethoxazole, Dapsone
- Atovaquoneantibiotics: Tetracycline*, Doxycycline, Erythromycin, Azythromycin, Clindamycin, Rifampicin, Trimethoprim
- Pentamidine

* Tetracycline eye ointments can be used.

# Annex II

**Signs or Symptoms Indicative of Severe Malaria**

- impaired consciousness, including unrousable coma (Blantyre Coma Score <5, GCS<15)
- prostration, i.e. generalized weakness so that the patient is unable to sit, stand or walk without assistance
- multiple convulsions: more than two episodes within 24h
- deep breathing and respiratory distress (acidotic breathing)
- acute pulmonary oedema and acute respiratory distress syndrome
- circulatory collapse or shock, systolic blood pressure
- < 80mm Hg in adults and < 50mm Hg in children
- acute kidney injury
- clinical jaundice plus evidence of other vital organ dysfunction
- Bleeding Disorder (Epistaxis, bleeding gums, frank haematuria)

For further reference see the Third Edition of the WHO publication “Management of severe malaria – A practical handbook”

<http://www.who.int/malaria/publications/atoz/9789241548526/en/index.html>

# Annex III

**Drug dosing for AL (Coartem)**

| **Drug** | **Day** | **Weight in kg** | | | |
| --- | --- | --- | --- | --- | --- |
|  |  | **6-14kg*** | **15-24kg** | **25-34kg** | **≥34+kg** |
| **Tab Coartem**  Arthemether 20 mg + Lumefantrine 120mg | **Day 0a**  **Day 0b** | 1  1 | 2  2 | 3  3 | 4  4 |
|  | **Day 1a**  **Day 1b** | 1  1 | 2  2 | 3  3 | 4  4 |
|  | **Day 2a**  **Day 2b** | 1  1 | 2  2 | 3  3 | 4  4 |

**For children under 5kg Coartem is not recommended according to the national guidelines (instead they receive Quinine 300mg 1/4 tablet for 7days). Children under 6kg (Revised malaria treatment Regimen 2009 DGHS) are excluded from this study.

# Annex IV

**Dosing schedule CQ**

Total dose - 25 mg/ kg body weight

| **Drug** | **Day** | **Weight in kg*** | | | | | |
| --- | --- | --- | --- | --- | --- | --- | --- |
|  |  | **6-9** | **10-19** | **20-29** | **30-39** | **40-49** | **50+** |
| **Tab CQ**  **150mg**  **Base** | **Day 0** | **1/2** | **1** | **1 1/2** | **2** | **3** | **4** |
|  | **Day 1** | **1/2** | **1** | **1 1/2** | **2** | **3** | **4** |
|  | **Day 2** | **1/2** | **1** | **1 1/2** | **2** | **2** | **2** |

*For children unable to swallow the tablets, they will be crushed in water. No syrup formulation available in Bangladesh

# Annex V

Primaquine (PQ) dose regimen

| **Drug** | **P.v. / P.f.** | **Day** | **Weight in kg** | | | | | |
| --- | --- | --- | --- | --- | --- | --- | --- | --- |
|  |  |  | **6-9** | **10-19** | **20-29** | **30-39** | **40-49** | **>50** |
| **Tab Primaquine 15 mg** | Treatment in P.v. cases | 3-16 | 1/4 | 1/2 | 1 | 1 | 1 | 1 |
|  | Treatment for P.f. cases | 3 (unsupervised) | 1/2 | 1 | 1 1/2 | 2 | 2 1/2 | 3 |

# Annex VI

Dosing of 2^nd^ line treatment and treatment for severe malaria

|  | drug | dosage |
| --- | --- | --- |
| P.f. 2^nd^ line treatment | Quinine (tablets) | 10mg/kg every 8 hours for 7 days |
| P.f. 3^rd^ line treatment | Quinine (tablets) + tetracycline | Q: 10mg/kg every 8 hours for 7 days  T: 250mg every 6 hours for 7 days |
|  | Quinine (tablets) + doxycycline | Q: 10mg/kg every 8 hours for 7 days  C: 100mg every 12 hours for 7 days |
| Severe malaria | Quinine IV/IM + quinine (tablets) | IV/IM: 10mg?kg followed by  Oral: Q: 10mg/kg every 8 hours for 7 days |
|  | Arthemether (IM) or artesunate (IV) followed by AL oral | 2.4mg/kg at H0, H12, H24 followed by Coartem daily for 3 days as indicated in Annex III |

# Annex VII

Definition of Treatment Failure

**Early treatment failure**

• Danger signs or severe malaria on day 1, 2 or 3 in the presence of peripheral parasitaemia;

• Parasitaemia on day 2 higher than on day 0, irrespective of axillary temperature;

• Parasitaemia on day 3 with axillary temperature ≥ 37.5 ºC;

• Parasitaemia on day 3 ≥ 25% of count on day 0.

**Late treatment failure**

**Late clinical failure**

• Danger signs or severe malaria in the presence of parasitaemia on any day between day 4 and last day of Follow up in patients who did not previously meet any of the criteria of early treatment failure;

• Presence of parasitaemia on any day between day 4 and the last day of follow up with axillary temperature ≥ 37.5 ºC (or history of fever) in patients who did not previously meet any of the criteria of early treatment failure

**Late parasitological failure**

Presence of parasitaemia on any day between day 7 and the last day of follow up with axillary temperature < 37.5 ºC in patients who did not previously meet any of the criteria of early treatment failure or late clinical failure

#

# Annex VIII

**International Centre for Diarrhoeal Disease Research, Bangladesh**

**Voluntary Consent Form (for aged 1-10 years)**

| **Protocol No. PR-14053** | **Version No. 1.6** | **Date: 09-09-2014** |
| --- | --- | --- |

Protocol Title: A study to assess primaquine treatment guidelines on malaria in south-east Bangladesh

Investigator’s name: Dr. Wasif Ali Khan

Organization: International Centre for Diarrhoeal Disease Research, Bangladesh

Sponsor: Menzies School of Health Research, Australia

**Introduction**

Malaria is a major cause of death worldwide and most cases occur in the poorest regions of the world. Malaria is caused by a parasite called “Plasmodium” that enters into the human body following the bite of female mosquitoes. Malaria is a major health problem in the mountainous Chittagong hill tract region of Bangladesh, where *Plasmodium falciparum* and *Plasmodium vivax* are two most common parasites causing malaria.

Malaria needs to be treated quickly and effectively. Bangladesh has a National Guidelines for treating malaria cases. Primaquine is a recommended drug by the guideline for treating malaria patients and is very good to ensure that the disease is fully cured. However the drug can cause a number of side effects in some people. A well known risk factor for side effects is among G6PD deficient individuals, a special form of blood cells that may result in a fast breakdown of blood cells when primaquine is given.

**Purpose of the research**

Current Bangladesh national treatment guideline recommends Primaquine as part of standard treatment procedures for malaria. Only one small study from Chittagong hill district showed around 1/5 to almost ½ of the tribal Bangladeshi population (20-40%) may be G6PD deficient and could possibly suffer from severe side effects of primaquine administration.

**Why have you been invited to participate in the study?**

Your child has been diagnosed to have malaria (falciparum or/and vivax) by the medical technologist in “Alikadam Upazila Health Complex”. He/ She will be treated according to the National Guidelines of Bangladesh in a moment by the duty physician. This is why we are inviting your child to participate in our study.

**Methods and procedures**

If you agree to let your child participate in this study :

1. We will collect data on his/her age, where you live and to what tribe you belong (if any).
2. We will do a quick examination of your child and record the findings of this examination.
3. We will then collect 4-5ml of blood from you. With the collected blood we:
   1. will check if he/she has anemia
   2. measure G6PD level of your child
   3. look at the genes that are responsible for your child’s G6PD form
   4. look at the malaria parasite genes to find out more about how effective the provided treatment really is

Some of these examinations will be done at the hospital, for others, your child’s blood sample will be transported to another laboratory.

1. We will contact your child a maximum of nine times in a period of a maximum of 30 days (day 1, 2, 3, 6, 9, 12, 16, 23 and 30), if he/she start to feel bad and this may be related to either the malaria infection or the treatment they have received, we would like you to come back with your child an additional time. During each visit we will ask how he/she feels and take a maximum of five drops of blood.
2. In case your child develops a very severe condition we have made arrangements that he/she will be transferred to the Chittagong Medical College Hospital where they will be managed with experience physicians. We will inform you and your doctor on any important finding we make during the physical examination of your child. If we discover at any point during the study period that your child has very severe side effects because of the drug, we will recommend to your doctor to change the treatment.
3. Severe G6PD deficient patients will not get primaquine and will be excluded from the study.

**Risk and benefits**

Your child may feel some pain and/or discomfort during collection of blood samples. There is a small chance of temporary bruising at the site of blood collection. We will follow aseptic procedures to minimize these risks as much as possible. In case of the rare event of an infection following blood collection, your child will be treated following standard procedures at no cost to yourself. No other compensation can be provided in the event of such a complication.

Your child’s participation in this study will help us to assess the safety of Primaquine in your community. Thus, your community will benefit as a whole from participation in this study. You or your child will not get any financial help for participating in the study.

**Privacy, anonymity and confidentiality**

We will not let anybody know that your child participated and we will not tell anybody about his/her test results. We will put your child’s name on a list and give him/her a unique number. Your child’s sample and all information provide can only be identified by this number. The principal investigator (Dr. Wasif Ali Khan) guarantees you that nobody outside of the study team and a monitoring body may see this list.

**Future use of information**

When we have finished this study we want to write an article about our findings. For this purpose study staff from the Menzies School of Health Research, Darwin, Australia, the University of California, San Francisco, USA and the International Centre for Diarrheal Disease Research, Bangladesh will look at the test results. Any results we have will be published without names or any information that will allow us to identify you or your child.

**Right not to participate and withdraw**

It is up to you whether your child will be in the study or not. You may choose not to allow your child to participate in the study. You can withdraw your child from the study any time after joining the study. It is your right to say at any point in time that you don’t want your child to be part of this study any longer. You can do so by telling any team member about this. You do not have to tell us why you changed your mind. Your child will not be treated any different and will receive exactly the same treatment at the hospital as anybody else. You do not have to pay any fees or penalties for this.

**Principle of compensation**

We will not pay you any money for the participation of your child in the study, nor will you receive any other benefit from this study, that you would not have received if your child not participated in the study.

**Answering your questions**

We will happily provide you further information about the study, now or at a later time point. You may communicate with the principal investigators of the study or his designated person via the contact address given below. We will try and answer all questions on any test performed in the course of this study, study objectives and procedures. However, we would like to inform you that some of the tests will be performed in batches (spectrophotometry), at weekly intervals, and thus the respective results will not be available immediately.

**Contact persons**

You are free to ask questions at any time. If later on you have additional questions about this study or in case of any injury or illness, you should contact study staff:

| Name of your Principal Investigator | **Dr. Wasif Ali Khan** |
| --- | --- |
| Address: | Centre for Vaccine Sciences , icddr,b, Mohakhali, Dhaka -1212 |
| 24 hours telephone number: | +880- 01730093257 |
| Office hours telephone number: | +880-2-9827057 |
| Name of study staff: | **Mr. Jacob Khyang** |
| Address: | icddr,b Bandarban Baptist church, Lushai Bari, Chimbuk Road, Bandarban |
| 24 hours telephone number: | 880-361-63-475(O), 01711-437553 |

If you have any complaints or questions about your child’s rights as a research volunteer, you may contact

| The name of the icddr,b IRB Contact: | **Mr. M.A. Salam Khan** |
| --- | --- |
| Address | IRB Secretariat, icddr,b, Mohakhali, Dhaka-1212  Cell: +880- 01711428989,  Office: +880-2-9827001-10 Ext. 3206, +880-2-9886498 |

I have read this consent form or someone explained it to me. I freely agree to allow my child to take part in the study.

Study code number:  *(affix ID code label here)*

Name of subject (print):

Name of the parent/guardian of minor subject (print):

_____________________________________ *_____________*

*Signature or fingerprint of the parent/guardian Date*

*of minor subject*

Name of the witness (print):

_____________________________________ *_____________*

*Signature of witness Date*

Name of the investigator/assigned person

who obtained consent (print):

_____________________________________ *_____________*

*Signature of the investigator/assigned Date*

*person who obtained consent*

**Second Step Consent**

From the blood test it has been found that your son/daughter has been suffering from mild/moderate type of G6PD deficiency disorder. We want to treat him/her with primaquine for 14 days. The treatment with primaquine may cause hemolysis (breakdown of your child red blood cells) as side effects of the drug. If it happens we will give necessary treatment for the hemolysis. If you now agree to be treated with primaquine then we will include your child in our study.

Name of subject (print):

Name of the parent/guardian of minor subject (print):

_____________________________________ *_____________*

*Signature or fingerprint of the parent/guardian Date*

*of minor subject*

Name of the witness (print):

_____________________________________ *_____________*

*Signature of witness Date*

Name of the investigator/assigned person

who obtained consent (print):

_____________________________________ *_____________*

*Signature of the investigator/assigned Date*

*person who obtained consent*

**International Centre for Diarrhoeal Disease Research, Bangladesh**

**Voluntary Consent Form (for adult)**

| **Protocol No. PR-14053** | **Version No. 1.6** | **Date: 09-09-2014** |
| --- | --- | --- |

Protocol Title: A study to assess primaquine treatment guidelines on malaria in south-east Bangladesh

Investigator’s name: Dr. Wasif Ali Khan

Organization: International Centre for Diarrhoeal Disease Research, Bangladesh

Sponsor: Menzies School of Health Research, Australia

**Introduction**

Malaria is a major cause of death worldwide and most cases occur in the poorest regions of the world. Malaria is caused by a parasite name called “Plasmodium” that enters into the human body following the bite of female mosquitoes. Malaria is a major health problem in the mountainous Chittagong hill tract region of Bangladesh, where *Plasmodium falciparum* and *Plasmodium vivax* are two most common parasites causing malaria.

Malaria needs to be treated quickly and effectively. Bangladesh has a National Guidelines for treating malaria cases. Primaquine is a recommended drug by the guideline for treating malaria patients and is very good to ensure that the disease is fully cured. However the drug can cause a number of side effects in some people. A well known risk factor for side effects is among G6PD deficient individuals, a special form of blood cells that may result in a fast breakdown of blood cells when primaquine is given.

**Purpose of the research**

Current Bangladesh national treatment guideline recommends Primaquine as part of standard treatment procedures for malaria. Only one small study from Chittagong hill district showed around 1/5 to almost ½ of the tribal Bangladeshi population (20-40%) may be G6PD deficient and could possibly suffer from severe side effects of primaquine administration.

**Why have you been invited to participate in the study?**

You have been diagnosed to have malaria (falciparum or/and vivax) by the medical technologist in “Alikadam Upazila Health Complex”. Malaria needs to be treated quickly and effectively and you will be treated according to the National Guidelines of Bangladesh in a moment by the duty physician. For this reason we would like to invite you participate in this study.

**Methods and procedures**

If you agree to participate in this study:

1. We will collect data on your age, where you live and to what tribe you belong (if any).
2. We will do a quick examination of your health and record the findings of this examination.
3. If you are a woman and between the age of 18 and 49 we will perform a pregnancy test. This is part of routine procedures and is done on all women of that age for this study.
4. We will then collect 5ml of blood from you. With the collected blood we:
   1. will check if you suffer from anemia
   2. measure your G6PD level
   3. look at the genes that are responsible for your G6PD form
   4. look at the malaria parasite genes to find out more about how effective the provided treatment really is

Some of these examinations will be done at the hospital, for others, your blood sample will be transported to another laboratory.

1. We will contact you a maximum of nine times in a period of a maximum of 30 days (day 1, 2, 3, 6, 9, 12, 16, 23 and 30), if you start to feel bad and this may be related to either the malaria infection or the treatment you have received, we would like you to come back an additional time. During each visit we will ask how you feel and take a maximum of five drops of blood.
2. In case you develop a very severe condition we have made arrangements that you will be transferred to the Chittagong Medical College Hospital where they will be managed with experience physicians.We will inform you and your doctor on any important finding we make during the physical examination. If we discover at any point during the study period that you have very severe side effects because of the drug, we will recommend to your doctor to change the treatment.
3. Severe G6PD deficient patients will not get primaquine and will be excluded from the study.

**Risk and benefits**

You may feel some pain and/or discomfort during collection of blood samples. There is a small chance of temporary bruising at the site of blood collection. We will follow aseptic procedures to minimize these risks as much as possible. In case of the rare event of an infection following blood collection, you will be treated following standard procedures at no cost to yourself. No other compensation can be provided in the event of such a complication.

Your participation in this study will help us to assess the safety of Primaquine in your community. Thus, the community will benefit as a whole through your participation in this study. You will not get any financial help for participating in the study.

**Privacy, anonymity and confidentiality**

We will not let anybody know that you participated and we will not tell anybody about your test results. We will put your name on a list and give you a unique number. Your sample and all information you provide can only be identified by this number. The principal investigator (Dr. Wasif Ali Khan) guarantees you that nobody outside of the study team and a monitoring body may see this list.

**Future use of information**

When we have finished this study we want to write an article about our findings. For this purpose study staff from the Menzies School of Health Research, Darwin, Australia, the University of California, San Francisco, USA and the International Centre for Diarrheal Disease Research, Bangladesh will look at the test results. Any results we have will be published without names or any information that will allow us to identify you or your family.

**Right not to participate and withdraw**

It is up to you whether you will be in the study or not. You may choose not to participate in the study. You can withdraw yourself from the study any time after joining the study. It is your right to say at any point in time that you don’t want to be part of this any longer. You can do so by telling any team member about this. You do not have to tell us why you changed your mind. You will not be treated any different and you will receive exactly the same treatment at the hospital as anybody else. You do not have to pay any fees or penalties.

**Principle of compensation**

We will not pay you any money for the participation of your child in the study, nor will you receive any other benefit from this study, that you would not have received if your child not participated in the study.

**Answering your questions**

We will happily provide you further information about the study, now or at a later time point. You may communicate with the principal investigators of the study or her/his designated person at the contact address given below. We will try and answer all your questions on any test performed in the course of this study, study objectives and study procedures. However, we would like to inform you that some of the tests will be performed in batches (spectrophotometry), at weekly intervals, and thus the respective results will not be available immediately.

**Contact persons**

You are free to ask questions at any time. If later on you have additional questions about this study or in case of any injury or illness, you should contact study staff:

| Name of your Principal Investigator | **Dr. Wasif Ali Khan** |
| --- | --- |
| Address: | Centre for Vaccine Sciences , icddr,b, Mohakhali, Dhaka -1212 |
| 24 hours telephone number: | +880- 01730093257 |
| Office hours telephone number: | +880-2-9827057 |
| Name of study staff: | **Mr. Jacob Khyang** |
| Address: | icddr,b Bandarban Baptist church, Lushai Bari, Chimbuk Road, Bandarban |
| 24 hours telephone number: | 880-361-63-475(O), 01711-437553 |

If you have any complaints or questions about your rights as a research volunteer, you may contact

| The name of the icddr,b IRB Contact: | **Mr. M.A. Salam Khan** |
| --- | --- |
| Address | IRB Secretariat, icddr,b, Mohakhali, Dhaka-1212  Cell: +880- 01711428989,  Office: +880-2-9827001-10 Ext. 3206, +880-2-9886498 |

I have read this consent form or someone explained it to me. I freely agree to be in the study.

Study code number:  *(affix ID code label here)*

Name of subject (print):

_____________________________________ *_____________*

*Signature or fingerprint of the subject Date*

Name of the witness (print):

_____________________________________ *_____________*

*Signature of witness Date*

Name of the investigator/assigned person

who obtained consent (print):

_____________________________________ *_____________*

*Signature of the investigator/assigned Date*

*person who obtained consent*

**Second Step Consent**

From the blood test it has been found that you are suffering from mild/moderate type of G6PD deficiency disorder. We want to treat you with primaquine for 14 days. The treatment with primaquine may cause hemolysis (breakdown of your red blood cells) as side effects of the drug. If it happens we will give necessary treatment for the hemolysis. If you now agree to be treated with primaquine then we will include you in our study.

Name of subject (print):

_____________________________________ *_____________*

*Signature or fingerprint of the subject Date*

Name of the witness (print):

_____________________________________ *_____________*

*Signature of witness Date*

Name of the investigator/assigned person

who obtained consent (print):

_____________________________________ *_____________*

*Signature of the investigator/assigned Date*

*person who obtained consent*

**International Centre for Diarrhoeal Disease Research, Bangladesh**

**Voluntary Assent Form (for aged 11-17 years)**

| **Protocol No. PR-14053** | **Version No. 1.6** | **Date: 09-09-2014** |
| --- | --- | --- |

Protocol Title: A study to assess primaquine treatment guidelines on malaria in south-east Bangladesh

Investigator’s name: Dr. Wasif Ali Khan

Organization: International Centre for Diarrhoeal Disease Research, Bangladesh

Sponsor: Menzies School of Health Research, Australia

**Introduction**

Malaria is a major cause of death worldwide and most cases occur in the poorest regions of the world. Malaria is caused by a parasite name called “Plasmodium” that enters into the human body following the bite of female mosquitoes. Malaria is a major health problem in the mountainous Chittagong hill tract region of Bangladesh, where *Plasmodium falciparum* and *Plasmodium vivax* are two most common parasites causing malaria.

Malaria needs to be treated quickly and effectively. Bangladesh has a National Guidelines for treating malaria cases. Primaquine is a recommended drug by the guideline for treating malaria patients and is very good to ensure that the disease is fully cured. However the drug can cause a number of side effects in some people. A well known risk factor for side effects is G6PD deficient individuals, a special form of blood cells that may result in a fast breakdown of blood cells when primaquine is given.

**Purpose of the research**

Current Bangladesh national treatment guideline recommends Primaquine as part of standard treatment procedures for malaria. Only one small study from Chittagong hill district showed around 1/5 to almost ½ of the tribal Bangladeshi population (20-40%) may be G6PD deficient and could possibly suffer from severe side effects of primaquine administration.

**Why have you been invited to participate in the study?**

You have been diagnosed to have malaria (falciparum or/and vivax) by the medical technologist in “Alikadam Upazila Health Complex”. Malaria needs to be treated quickly and effectively and you will be treated according to the National Guidelines of Bangladesh in a moment by the duty physician. For this reason we would like to invite you participate in this study.

**Methods and procedures**

If you agree to participate in this study:

1. We will collect data on your age, where you live and to what tribe you belong (if any).
2. We will do a quick examination of your health and record the findings of this examination.
3. If you are a girl and between the age of 13 and 17 we will perform a pregnancy test. This is part of routine procedures and is done on all girls of that age for this study.
4. We will then collect 5ml of blood from you. With the collected blood we:
   1. will check if you suffer from anemia
   2. measure your G6PD level
   3. look at the genes that are responsible for your G6PD form
   4. look at the malaria parasite genes to find out more about how effective the provided treatment really is.

Some of these examinations will be done at the hospital, for others, your sample will be transported to another laboratory.

1. We will contact you a maximum of nine times in a period of a maximum of 30 days (day 1, 2, 3, 6, 9, 12, 16, 23 and 30), if you start to feel bad and this may be related to either the malaria infection or the treatment you have received, we would like you to come back an additional time. During each visit we will ask how you feel and take a maximum of five drops of blood.
2. In case you develop a very severe condition we have made arrangements that you will be transferred to the Chittagong Medical College. We will inform you and your doctor on any important finding we make during the physical examination. If we discover at any point during the study period that you have very severe side effects because of the drug, we will recommend to your doctor to change the treatment.
3. Severe G6PD deficient patients will not get primaquine and will be excluded from the study.

**Risk and benefits**

You may feel some pain and/or discomfort during collection of blood samples. There is a small chance of temporary bruising at the site of blood collection. We will follow aseptic procedures to minimize these risks as much as possible. In case of the rare event of an infection following blood collection, you will be treated following standard procedures at no cost to yourself. No other compensation can be provided in the event of such a complication.

Your participation in this study will help us to assess the safety of Primaquine in your community. Thus, the community will benefit as a whole through your participation in this study. You will not get any financial help for participating in the study.

**Privacy, anonymity and confidentiality**

We will not let anybody know that you participated and we will not tell anybody about your test results. We will put your name on a list and give you a unique number. Your sample and all information you provide can only be identified by this number. The principal investigator (Dr. Wasif Ali Khan) guarantees you that nobody outside of the study team and a monitoring body may see this list.

**Future use of information**

When we have finished this study we want to write an article about our findings. For this purpose study staff from the Menzies School of Health Research, Darwin, Australia, the University of California, San Francisco, USA and the International Centre for Diarrheal Disease Research, Bangladesh will look at the test results. Any results we have will be published without names or any information that will allow us to identify you or your family.

**Right not to participate and withdraw**

It is up to you whether you will be in the study or not. You may choose not to participate in the study. You can withdraw yourself from the study any time after joining the study. It is your right to say at any point in time that you don’t want to be part of this any longer. You can do so by telling any team member about this. You do not have to tell us why you changed your mind. You will not be treated any different and you will receive exactly the same treatment at the hospital as anybody else. You do not have to pay any fees or penalties.

**Principle of compensation**

We will not pay you any money for the participation of your child in the study, nor will you receive any other benefit from this study, that you would not have received if your child not participated in the study.

**Answering your questions**

We will happily provide you further information about the study, now or at a later time point. You may communicate with the principal investigators of the study or her/his designated person at the contact address given below. We will try and answer all your questions on any test performed in the course of this study, study objectives and study procedures. However, we would like to inform you that some of the tests will be performed in batches (spectrophotometry), at weekly intervals, and thus the respective results will not be available immediately.

**Contact persons**

You are free to ask questions at any time. If later on you have additional questions about this study or in case of any injury or illness, you should contact study staff:

| Name of your Principal Investigator | **Dr. Wasif Ali Khan** |
| --- | --- |
| Address: | Centre for Vaccine Sciences , icddr,b, Mohakhali, Dhaka -1212 |
| 24 hours telephone number: | +880- 01730093257 |
| Office hours telephone number: | +880-2-9827057 |
| Name of study staff: | **Mr. Jacob Khyang** |
| Address: | icddr,b Bandarban Baptist church, Lushai Bari, Chimbuk Road, Bandarban |
| 24 hours telephone number: | 880-361-63-475(O), 01711-437553 |

If you have any complaints or questions about your rights as a research volunteer, you may contact

| The name of the icddr,b IRB Contact: | **Mr. M.A. Salam Khan** |
| --- | --- |
| Address | IRB Secretariat, icddr,b, Mohakhali, Dhaka-1212  Cell: +880- 01711428989,  Office: +880-2-9827001-10 Ext. 3206, +880-2-9886498 |

I have read this consent form or someone explained it to me. I freely agree to be in the study.

Study code number:  *(affix ID code label here)*

Name of subject (print):

Verbal consent for children 11-17 years obtained Yes No

Name of the parent/guardian of the subject (print):

_____________________________________ *_____________*

*Signature or fingerprint of the parent/guardian Date*

*of the subject*

Name of the witness (print):

_____________________________________ *_____________*

*Signature of witness Date*

Name of the investigator/assigned person

who obtained consent (print):

_____________________________________ *_____________*

*Signature of the investigator/assigned Date*

*person who obtained consent*

**Second Step Assent**

From the blood test it has been found that you are suffering from mild/moderate type of G6PD deficiency disorder. We want to treat you with primaquine for 14 days. The treatment with primaquine may cause hemolysis (breakdown of your red blood cells) as side effects of the drug. If it happens we will give necessary treatment for the hemolysis. If you now agree to be treated with primaquine then we will include you in our study.

Name of subject (print):

Verbal consent for children 11-17 years obtained Yes No

Name of the parent/guardian of the subject (print):

_____________________________________ *_____________*

*Signature or fingerprint of the parent/guardian Date*

*of the subject*

Name of the witness (print):

_____________________________________ *_____________*

*Signature of witness Date*

Name of the investigator/assigned person

who obtained consent (print):

_____________________________________ *_____________*

*Signature of the investigator/assigned Date*

*person who obtained consent*

**Annex IX**

**Case record form**

**Date of Birth:** **OR** **Age:** ☐☐ in ☐ Month ☐ Year

D

D

M

M

Y

Y

Y

Y

**Sex:** ☐ Male ☐ Female **Ethnicity:** ☐ Tribal ☐ Non Tribal

**If tribal**, which tribe? ☐ Marma ☐ Chakma ☐ Tripura ☐ Tanchangya ☐ Khyang ☐ Bawm ☐ Rakhaine ☐ Mro ☐ Pangkho ☐ Lusai ☐ Khumi ☐ Chak ☐ Other (specify) _______________________

**Day – 0 Date of visit:** ☐☐-☐☐-☐☐☐☐

1. **Weight:** ☐☐☐.☐ Kg

2. **Temperature:** ☐☐.☐ ˚C

3. **Finger prick (baseline) collected?** ☐ Yes ☐ No

*If yes:* **Blood slide (baseline) collected?** ☐ Yes ☐ No

*If yes:* Time: ☐☐:☐☐ in 24:00 format (e.g. 4pm is 16:00)

**Parasite count (baseline):** ________/500RBC or ___________/200WBC

**Diagnosis:** ☐ Pf ☐ Pv ☐Mixed

*If yes:* **Hemoglobin (baseline) measured?** ☐ Yes ☐ No

*if Yes:* ☐☐.☐ g/dl

4. **Venous blood (baseline) collected?** ☐ Yes ☐

5. **Does this patient have a family history of hemolysis (severe anemia)?** ☐ Yes ☐ No ☐Uncertain

*If yes, was it in response to:*  ☐Medication ☐Food ☐Infection ☐Other

6. **Does this patient have a family history of G6PD deficiency?** ☐ Yes ☐No ☐ Uncertain

7. **Has this patient ever had a blood transfusion?** ☐ Yes ☐ No

*If yes, within the last 12 weeks?* ☐ *Yes* ☐ *No* ☐ *Uncertain*

8. **2^nd^ Blood slide collected?** ☐ Yes ☐ No

*If yes:* Time: ☐☐:☐☐ in 24:00 format (e.g. 4pm is 16:00)

**Parasite count:** ________/500RBC or ___________/200WBC

**Diagnosis:** ☐ Pf ☐ Pv ☐Mixed

9. **Schizontocidal Treatment**

| Schizontocidal treatment | Pf | Pv | mixed |
| --- | --- | --- | --- |
| 1^st^ Dose (observed) | AL given  ☐ Yes ☐ No ☐  Dose:___________ | CQ given  ☐ Yes ☐ No ☐  Dose:___________ | AL given  ☐ Yes ☐ No ☐  Dose:___________ |
| 2^nd^ Dose | AL given  ☐ Yes ☐ No ☐  Dose:___________ | N/A | AL given  ☐ Yes ☐ No ☐  Dose:___________ |
|  | Was this dose given as observed treatment  ☐yes ☐no |  | Was this dose given as observed treatment  ☐yes ☐no |

**Day – 1 Date of visit:** ☐☐-☐☐-☐☐☐☐

10. **Temperature:** ☐☐ **.** ☐˚C

11. **Finger prick collected?** ☐ Yes ☐ No

*If yes:* **Blood slide collected?** ☐ Yes ☐ No

*If yes:* Time: ☐☐:☐☐ in 24:00 format (e.g. 4pm is 16:00)

**Parasite count:** ________/500RBC or ___________/200WBC

*If yes:* **Hemoglobin measured?** ☐ Yes ☐ No

*if Yes:* ☐☐.☐ g/dl

12. **2^nd^ Blood slide collected?** ☐ Yes ☐ No

*If yes:* Time: ☐☐:☐☐ in 24:00 format (e.g. 4pm is 16:00)

**Parasite count :**________/500RBC or ___________/200WBC

13. **Schizontocidal Treatment**

| Schizontocidal treatment | Pf | Pv | mixed |
| --- | --- | --- | --- |
| 1^st^ Dose (observed) | AL given  ☐ Yes ☐ No ☐  Dose:___________ | CQ given  ☐ Yes ☐ No ☐  Dose:___________ | AL given  ☐ Yes ☐ No ☐  Dose:___________ |
| 2^nd^ Dose | AL given  ☐ Yes ☐ No ☐  Dose:___________ | N/A | AL given  ☐ Yes ☐ No ☐  Dose:___________ |
|  | Was this dose given as observed treatment  ☐yes ☐no |  | Was this dose given as observed treatment  ☐yes ☐no |

**Day – 2: Date of visit:** ☐☐-☐☐-☐☐☐☐

14. **Temperature:** ☐☐.☐˚C

15. **Finger prick collected?** ☐ Yes ☐ No

*If yes:* **Blood slide collected?** ☐ Yes ☐ No

*If yes:* Time: ☐☐:☐☐ in 24:00 format (e.g. 4pm is 16:00)

**Parasite count** ________/500RBC or ___________/200WBC

*If yes:* **Hemoglobin measured?** ☐ Yes ☐ No

*if Yes:* ☐☐.☐ g/dl

16. **2^nd^ Blood slide collected?** ☐ Yes ☐ No

*If yes:* Time: ☐☐:☐☐ in 24:00 format (e.g. 4pm is 16:00)

**Parasite count** ________/500RBC or ___________/200WBC

**17. G6PD result:** ☐☐.☐ **IU/g Hb**

18. **Schizontocidal Treatment**

| Schizontocidal treatment | Pf | Pv | mixed |
| --- | --- | --- | --- |
| 1^st^ Dose (observed) | AL given  ☐ Yes ☐ No ☐  Dose:___________ | CQ given  ☐ Yes ☐ No ☐  Dose:___________ | AL given  ☐ Yes ☐ No ☐  Dose:___________ |
| 2^nd^ Dose | AL given  ☐ Yes ☐ No ☐  Dose:___________ | N/A | AL given  ☐ Yes ☐ No ☐  Dose:___________ |
|  | Was this dose given as observed treatment  ☐yes ☐no |  | Was this dose given as observed treatment  ☐yes ☐no |

19. **Primaquine treatment**

| Primaquine treatment | Pf | Pv | mixed |
| --- | --- | --- | --- |
|  | PQ single dose provided tp patient  ☐ Yes ☐ No ☐  Dose:___________ | PQ full course provided to patient  ☐ Yes ☐ No ☐  Dose:___________ | PQ full course provided to patient  ☐ Yes ☐ No ☐  Dose:___________ |

**Day – 6: Date of visit:** ☐☐-☐☐-☐☐☐☐

20. **Temperature:** ☐☐.☐˚C

21. **Finger prick collected?** ☐ Yes ☐ No

*If yes:* **Blood slide collected?** ☐ Yes ☐ No

*If yes:* Time: ☐☐:☐☐ in 24:00 format (e.g. 4pm is 16:00)

**Parasite count** ________/500RBC or ___________/200WBC

Diagnosis: ☐ Pf ☐ Pv ☐Mixed ☐neg

*If yes:* **Hemoglobin measured?** ☐ Yes ☐ No

*if Yes:* ☐☐.☐ g/dl

**Day – 9: Date of visit:** ☐☐-☐☐-☐☐☐☐

22. **Temperature:** ☐☐.☐˚C

23. **Finger prick collected?** ☐ Yes ☐ No

*If yes:* **Blood slide collected?** ☐ Yes ☐ No

*If yes:* Time: ☐☐:☐☐ in 24:00 format (e.g. 4pm is 16:00)

**Parasite count:** ________/500RBC or ___________/200WBC

Diagnosis: ☐ Pf ☐ Pv ☐Mixed ☐neg

*If yes:* **Hemoglobin measured?** ☐ Yes ☐ No

*if Yes:* ☐☐.☐ g/dl

**Day – 12: Date of visit:** ☐☐-☐☐-☐☐☐☐

24. **Temperature:** ☐☐.☐˚C

25. **Finger prick collected?** ☐ Yes ☐ No

*If yes:* **Blood slide collected?** ☐ Yes ☐ No

*If yes:* Time: ☐☐:☐☐ in 24:00 format (e.g. 4pm is 16:00)

**Parasite count :** ________/500RBC or ___________/200WBC

Diagnosis: ☐ Pf ☐ Pv ☐Mixed ☐neg

*If yes:* **Hemoglobin measured?** ☐ Yes ☐ No

*if Yes:* ☐☐.☐ g/dl

**Day – 16: Date of visit:** ☐☐-☐☐-☐☐☐☐

26. **Temperature:** ☐☐.☐˚C

27. **Finger prick collected?** ☐ Yes ☐ No

*If yes:* **Blood slide collected?** ☐ Yes ☐ No

*If yes:* Time: ☐☐:☐☐ in 24:00 format (e.g. 4pm is 16:00)

**Parasite count :** ________/500RBC or ___________/200WBC

Diagnosis: ☐ Pf ☐ Pv ☐Mixed ☐neg

*If yes:* **Hemoglobin measured?** ☐ Yes ☐ No

*if Yes:* ☐☐.☐ g/dl

28. **Meth hemoglobin measured (only Pf/Pv and Pv patients > 2 years)?** ☐ Yes ☐ No

*if Yes:* ☐☐.☐ in %

29. **# of days PQ tablets taken:** ___________________________________________

30**. Remaining tablets:** _____________________________________________ ☐Don’t known

**Day – 23: Date of visit:** ☐☐-☐☐-☐☐☐☐

24. **Temperature:** ☐☐.☐˚C

25. **Finger prick collected?** ☐ Yes ☐ No

*If yes:* **Blood slide collected?** ☐ Yes ☐ No

*If yes:* Time: ☐☐:☐☐ in 24:00 format (e.g. 4pm is 16:00)

**Parasite count :** ________/500RBC or ___________/200WBC

Diagnosis: ☐ Pf ☐ Pv ☐Mixed ☐neg

*If yes:* **Hemoglobin measured?** ☐ Yes ☐ No

*if Yes:* ☐☐.☐ g/dl

**Day – 30: Date of visit:** ☐☐-☐☐-☐☐☐☐

24. **Temperature:** ☐☐.☐˚C

25. **Finger prick collected?** ☐ Yes ☐ No

*If yes:* **Blood slide collected?** ☐ Yes ☐ No

*If yes:* Time: ☐☐:☐☐ in 24:00 format (e.g. 4pm is 16:00)

**Parasite count :** ________/500RBC or ___________/200WBC

Diagnosis: ☐ Pf ☐ Pv ☐Mixed ☐neg

*If yes:* **Hemoglobin measured?** ☐ Yes ☐ No

*if Yes:* ☐☐.☐ g/dl

**1^st^ Unscheduled visit: Date of visit:** ☐☐-☐☐-☐☐☐☐

31. **Temperature:** ☐☐.☐˚C

32. **Finger prick collected?** ☐ Yes ☐ No

*If yes:* **Blood slide collected?** ☐ Yes ☐ No

*If yes:* Time: ☐☐:☐☐ in 24:00 format (e.g. 4pm is 16:00)

**Parasite count :** ________/500RBC or ___________/200WBC

Diagnosis: ☐ Pf ☐ Pv ☐Mixed ☐neg

*If yes:* **Hemoglobin measured?** ☐ Yes ☐ No

*if Yes:* ☐☐.☐ g/dl

33. **Meth hemoglobin measured (only Pf/Pv and Pv patients > 2 years)?** ☐ Yes ☐ No

*if Yes:* ☐☐.☐ in %

**2^nd^ Unscheduled visit: Date of visit:** ☐☐-☐☐-☐☐☐☐

34. **Temperature:** ☐☐.☐˚C

35. **Finger prick collected?** ☐ Yes ☐ No

*If yes:* **Blood slide collected?** ☐ Yes ☐ No

*If yes:* Time: ☐☐:☐☐ in 24:00 format (e.g. 4pm is 16:00)

**Parasite count :** ________/500RBC or ___________/200WBC

Diagnosis: ☐ Pf ☐ Pv ☐Mixed ☐neg

*If yes:* **Hemoglobin measured?** ☐ Yes ☐ No

*if Yes:* ☐☐.☐ g/dl

36. **Meth hemoglobin measured (only Pf/Pv and Pv patients > 2 years)?** ☐ Yes ☐ No

*if Yes:* ☐☐.☐ in %

**SYMPTOM REPORTING FORM - Page 1**

| **Type of AE**  **(one per row only)** | **Onset Date** | **Severity Grade**  **(check one)** | **Actions Taken?**  **(specify below if needed)** | **Is AE related to PQ?**  **(check one)** | **Date Resolved** | **Outcome of AE (check one)** |
| --- | --- | --- | --- | --- | --- | --- |
| ☐Rash  ☐Nausea  ☐Vomiting  ☐Abdominal pain  ☐Dark Urine  Hillmen index: _ _  ☐Diarrhea  ☐Poor appetite  ☐Other (describe): _________________ | _ _ / _ _ / _ _ | ☐ Mild  ☐ Moderate  ☐Severe  ☐ Life-threatening | ☐ None  ☐ Stopped PQ  ☐ Prescribed drug  ☐ Blood Transfusion  ☐ Hospitalization  ☐ Other** | ☐ Unrelated  ☐ Unlikely  ☐ Possible  ☐ Probable  ☐ Definite | _ _ / _ _ / _ _  or ongoing ☐ | ☐ Recovered/resolved  ☐ Recovered/resolved with sequelae  ☐ Recovering/resolving  ☐ Not recovered/not resolved  ☐ Fatal  ☐ Unknown |
| ☐Rash  ☐Nausea  ☐Vomiting  ☐Abdominal pain  ☐Dark Urine  Hillmen index: _ _  ☐Diarrhea  ☐Poor appetite  ☐Other (describe): _________________ | _ _ / _ _ / _ _ | ☐ Mild  ☐ Moderate  ☐Severe  ☐ Life-threatening | ☐ None  ☐ Stopped PQ  ☐ Prescribed drug  ☐ Blood Transfusion  ☐ Hospitalization  ☐ Other** | ☐ Unrelated  ☐ Unlikely  ☐ Possible  ☐ Probable  ☐ Definite | _ _ / _ _ / _ _  or ongoing ☐ | ☐ Recovered/resolved  ☐ Recovered/resolved with sequelae  ☐ Recovering/resolving  ☐ Not recovered/not resolved  ☐ Fatal  ☐ Unknown |
| **Definitions**  Severity – Mild (easily tolerated; no or minimal interference with daily activities); Moderate (low level of inconvenience; greater than minimal interference with daily activities); Severe (interrupts normal daily activities; usually incapacitating); Life-threatening (life-threatening consequences; urgent intervention indicated); Fatal (death) | | | | | | |

**SYMPTOM REPORTING FORM - Page 2**

| **Type of AE**  **(one per row only)** | **Onset Date** | **Severity Grade**  **(check one)** | **Actions Taken?**  **(specify below if needed)** | **Is AE related to PQ?**  **(check one)** | **Date Resolved** | **Outcome of AE (check one)** |
| --- | --- | --- | --- | --- | --- | --- |
| ☐Rash  ☐Nausea  ☐Vomiting  ☐Abdominal pain  ☐Dark Urine  Hillmen index: _ _  ☐Diarrhea  ☐Poor appetite  ☐Other (describe): _________________ | _ _ / _ _ / _ _ | ☐ Mild  ☐ Moderate  ☐Severe  ☐ Life-threatening | ☐ None  ☐ Stopped PQ  ☐ Prescribed drug  ☐ Blood Transfusion  ☐ Hospitalization  ☐ Other** | ☐ Unrelated  ☐ Unlikely  ☐ Possible  ☐ Probable  ☐ Definite | _ _ / _ _ / _ _  or ongoing ☐ | ☐ Recovered/resolved  ☐ Recovered/resolved with sequelae  ☐ Recovering/resolving  ☐ Not recovered/not resolved  ☐ Fatal  ☐ Unknown |
| ☐Rash  ☐Nausea  ☐Vomiting  ☐Abdominal pain  ☐Dark Urine  Hillmen index: _ _  ☐Diarrhea  ☐Poor appetite  ☐Other (describe): _________________ | _ _ / _ _ / _ _ | ☐ Mild  ☐ Moderate  ☐Severe  ☐ Life-threatening | ☐ None  ☐ Stopped PQ  ☐ Prescribed drug  ☐ Blood Transfusion  ☐ Hospitalization  ☐ Other** | ☐ Unrelated  ☐ Unlikely  ☐ Possible  ☐ Probable  ☐ Definite | _ _ / _ _ / _ _  or ongoing ☐ | ☐ Recovered/resolved  ☐ Recovered/resolved with sequelae  ☐ Recovering/resolving  ☐ Not recovered/not resolved  ☐ Fatal  ☐ Unknown |
| **Definitions**  Severity – Mild (easily tolerated; no or minimal interference with daily activities); Moderate (low level of inconvenience; greater than minimal interference with daily activities); Severe (interrupts normal daily activities; usually incapacitating); Life-threatening (life-threatening consequences; urgent intervention indicated); Fatal (death) | | | | | | |

**SYMPTOM REPORTING FORM - Page 3**

| **Type of AE**  **(one per row only)** | **Onset Date** | **Severity Grade**  **(check one)** | **Actions Taken?**  **(specify below if needed)** | **Is AE related to PQ?**  **(check one)** | **Date Resolved** | **Outcome of AE (check one)** |
| --- | --- | --- | --- | --- | --- | --- |
| ☐Rash  ☐Nausea  ☐Vomiting  ☐Abdominal pain  ☐Dark Urine  Hillmen index: _ _  ☐Diarrhea  ☐Poor appetite  ☐Other (describe): _________________ | _ _ / _ _ / _ _ | ☐ Mild  ☐ Moderate  ☐Severe  ☐ Life-threatening | ☐ None  ☐ Stopped PQ  ☐ Prescribed drug  ☐ Blood Transfusion  ☐ Hospitalization  ☐ Other** | ☐ Unrelated  ☐ Unlikely  ☐ Possible  ☐ Probable  ☐ Definite | _ _ / _ _ / _ _  or ongoing ☐ | ☐ Recovered/resolved  ☐ Recovered/resolved with sequelae  ☐ Recovering/resolving  ☐ Not recovered/not resolved  ☐ Fatal  ☐ Unknown |
| ☐Rash  ☐Nausea  ☐Vomiting  ☐Abdominal pain  ☐Dark Urine  Hillmen index: _ _  ☐Diarrhea  ☐Poor appetite  ☐Other (describe): _________________ | _ _ / _ _ / _ _ | ☐ Mild  ☐ Moderate  ☐Severe  ☐ Life-threatening | ☐ None  ☐ Stopped PQ  ☐ Prescribed drug  ☐ Blood Transfusion  ☐ Hospitalization  ☐ Other** | ☐ Unrelated  ☐ Unlikely  ☐ Possible  ☐ Probable  ☐ Definite | _ _ / _ _ / _ _  or ongoing ☐ | ☐ Recovered/resolved  ☐ Recovered/resolved with sequelae  ☐ Recovering/resolving  ☐ Not recovered/not resolved  ☐ Fatal  ☐ Unknown |
| **Definitions**  Severity – Mild (easily tolerated; no or minimal interference with daily activities); Moderate (low level of inconvenience; greater than minimal interference with daily activities); Severe (interrupts normal daily activities; usually incapacitating); Life-threatening (life-threatening consequences; urgent intervention indicated); Fatal (death) | | | | | | |

**SYMPTOM REPORTING FORM - Page 4**

| **Type of AE**  **(one per row only)** | **Onset Date** | **Severity Grade**  **(check one)** | **Actions Taken?**  **(specify below if needed)** | **Is AE related to PQ?**  **(check one)** | **Date Resolved** | **Outcome of AE (check one)** |
| --- | --- | --- | --- | --- | --- | --- |
| ☐Rash  ☐Nausea  ☐Vomiting  ☐Abdominal pain  ☐Dark Urine  Hillmen index: _ _  ☐Diarrhea  ☐Poor appetite  ☐Other (describe): _________________ | _ _ / _ _ / _ _ | ☐ Mild  ☐ Moderate  ☐Severe  ☐ Life-threatening | ☐ None  ☐ Stopped PQ  ☐ Prescribed drug  ☐ Blood Transfusion  ☐ Hospitalization  ☐ Other** | ☐ Unrelated  ☐ Unlikely  ☐ Possible  ☐ Probable  ☐ Definite | _ _ / _ _ / _ _  or ongoing ☐ | ☐ Recovered/resolved  ☐ Recovered/resolved with sequelae  ☐ Recovering/resolving  ☐ Not recovered/not resolved  ☐ Fatal  ☐ Unknown |
| ☐Rash  ☐Nausea  ☐Vomiting  ☐Abdominal pain  ☐Dark Urine  Hillmen index: _ _  ☐Diarrhea  ☐Poor appetite  ☐Other (describe): _________________ | _ _ / _ _ / _ _ | ☐ Mild  ☐ Moderate  ☐Severe  ☐ Life-threatening | ☐ None  ☐ Stopped PQ  ☐ Prescribed drug  ☐ Blood Transfusion  ☐ Hospitalization  ☐ Other** | ☐ Unrelated  ☐ Unlikely  ☐ Possible  ☐ Probable  ☐ Definite | _ _ / _ _ / _ _  or ongoing ☐ | ☐ Recovered/resolved  ☐ Recovered/resolved with sequelae  ☐ Recovering/resolving  ☐ Not recovered/not resolved  ☐ Fatal  ☐ Unknown |
| **Definitions**  Severity – Mild (easily tolerated; no or minimal interference with daily activities); Moderate (low level of inconvenience; greater than minimal interference with daily activities); Severe (interrupts normal daily activities; usually incapacitating); Life-threatening (life-threatening consequences; urgent intervention indicated); Fatal (death) | | | | | | |

**SYMPTOM REPORTING FORM - Page 5**

| **Type of AE**  **(one per row only)** | **Onset Date** | **Severity Grade**  **(check one)** | **Actions Taken?**  **(specify below if needed)** | **Is AE related to PQ?**  **(check one)** | **Date Resolved** | **Outcome of AE (check one)** |
| --- | --- | --- | --- | --- | --- | --- |
| ☐Rash  ☐Nausea  ☐Vomiting  ☐Abdominal pain  ☐Dark Urine  Hillmen index: _ _  ☐Diarrhea  ☐Poor appetite  ☐Other (describe): _________________ | _ _ / _ _ / _ _ | ☐ Mild  ☐ Moderate  ☐Severe  ☐ Life-threatening | ☐ None  ☐ Stopped PQ  ☐ Prescribed drug  ☐ Blood Transfusion  ☐ Hospitalization  ☐ Other** | ☐ Unrelated  ☐ Unlikely  ☐ Possible  ☐ Probable  ☐ Definite | _ _ / _ _ / _ _  or ongoing ☐ | ☐ Recovered/resolved  ☐ Recovered/resolved with sequelae  ☐ Recovering/resolving  ☐ Not recovered/not resolved  ☐ Fatal  ☐ Unknown |
| ☐Rash  ☐Nausea  ☐Vomiting  ☐Abdominal pain  ☐Dark Urine  Hillmen index: _ _  ☐Diarrhea  ☐Poor appetite  ☐Other (describe): _________________ | _ _ / _ _ / _ _ | ☐ Mild  ☐ Moderate  ☐Severe  ☐ Life-threatening | ☐ None  ☐ Stopped PQ  ☐ Prescribed drug  ☐ Blood Transfusion  ☐ Hospitalization  ☐ Other** | ☐ Unrelated  ☐ Unlikely  ☐ Possible  ☐ Probable  ☐ Definite | _ _ / _ _ / _ _  or ongoing ☐ | ☐ Recovered/resolved  ☐ Recovered/resolved with sequelae  ☐ Recovering/resolving  ☐ Not recovered/not resolved  ☐ Fatal  ☐ Unknown |
| **Definitions**  Severity – Mild (easily tolerated; no or minimal interference with daily activities); Moderate (low level of inconvenience; greater than minimal interference with daily activities); Severe (interrupts normal daily activities; usually incapacitating); Life-threatening (life-threatening consequences; urgent intervention indicated); Fatal (death)  ****Other information (actions taken): ___________________________________________________________________________________**  **___________________________________________________________________________________________________________________** | | | | | | |

**Concomitant medication Table Page 1 (only if any symptoms with grading 3 or 4 occurred)**

| **Name of Drug**  **(please use a new row for each drug)** | **Dose** | **Unit** | **Freq (number of times per day)** | **Route** | **Date Started (dd/mm/yy)** | **Date Stopped (dd/mm/yy)**  **Or Continuing?** | **Indication**  **(please make sure that this is in accordance with the symptoms table)** |
| --- | --- | --- | --- | --- | --- | --- | --- |
|  |  | ☐ mg  ☐ g  ☐ ml  ☐ mcg  ☐ drops  ☐tsp  ☐tablet/capsule  ☐don’t know  ☐ other:_____ |  | ☐ oral  ☐ topical  ☐ IM  ☐ rectal  ☐ IV  ☐ don’t know  ☐ other : ________ | _ _ / _ _ / _ _ | _ _ / _ _ / _ _  ☐Continuing |  |
|  |  | ☐ mg  ☐ g  ☐ ml  ☐ mcg  ☐ drops  ☐tsp  ☐tablet/capsule  ☐don’t know  ☐ other:_____ |  | ☐ oral  ☐ topical  ☐ IM  ☐ rectal  ☐ IV  ☐ don’t know  ☐ other : ________ | _ _ / _ _ / _ _ | _ _ / _ _ / _ _  ☐Continuing |  |
|  |  | ☐ mg  ☐ g  ☐ ml  ☐ mcg  ☐ drops  ☐tsp  ☐tablet/capsule  ☐don’t know  ☐ other:_____ |  | ☐ oral  ☐ topical  ☐ IM  ☐ rectal  ☐ IV  ☐ don’t know  ☐ other : ________ | _ _ / _ _ / _ _ | _ _ / _ _ / _ _  ☐Continuing |  |

**Concomitant medication Table Page 2 (only if any symptoms with grading 3 or 4 occurred)**

| **Name of Drug**  **(please use a new row for each drug)** | **Dose** | **Unit** | **Freq (number of times per day)** | **Route** | **Date Started (dd/mm/yy)** | **Date Stopped (dd/mm/yy)**  **Or Continuing?** | **Indication**  **(please make sure that this is in accordance with the symptoms table)** |
| --- | --- | --- | --- | --- | --- | --- | --- |
|  |  | ☐ mg  ☐ g  ☐ ml  ☐ mcg  ☐ drops  ☐tsp  ☐tablet/capsule  ☐don’t know  ☐ other:_____ |  | ☐ oral  ☐ topical  ☐ IM  ☐ rectal  ☐ IV  ☐ don’t know  ☐ other : ________ | _ _ / _ _ / _ _ | _ _ / _ _ / _ _  ☐Continuing |  |
|  |  | ☐ mg  ☐ g  ☐ ml  ☐ mcg  ☐ drops  ☐tsp  ☐tablet/capsule  ☐don’t know  ☐ other:_____ |  | ☐ oral  ☐ topical  ☐ IM  ☐ rectal  ☐ IV  ☐ don’t know  ☐ other : ________ | _ _ / _ _ / _ _ | _ _ / _ _ / _ _  ☐Continuing |  |
|  |  | ☐ mg  ☐ g  ☐ ml  ☐ mcg  ☐ drops  ☐tsp  ☐tablet/capsule  ☐don’t know  ☐ other:_____ |  | ☐ oral  ☐ topical  ☐ IM  ☐ rectal  ☐ IV  ☐ don’t know  ☐ other : ________ | _ _ / _ _ / _ _ | _ _ / _ _ / _ _  ☐Continuing |  |

**Concomitant medication Table Page 3 (only if any symptoms with grading 3 or 4 occurred)**

| **Name of Drug**  **(please use a new row for each drug)** | **Dose** | **Unit** | **Freq (number of times per day)** | **Route** | **Date Started (dd/mm/yy)** | **Date Stopped (dd/mm/yy)**  **Or Continuing?** | **Indication**  **(please make sure that this is in accordance with the symptoms table)** |
| --- | --- | --- | --- | --- | --- | --- | --- |
|  |  | ☐ mg  ☐ g  ☐ ml  ☐ mcg  ☐ drops  ☐tsp  ☐tablet/capsule  ☐don’t know  ☐ other:_____ |  | ☐ oral  ☐ topical  ☐ IM  ☐ rectal  ☐ IV  ☐ don’t know  ☐ other : ________ | _ _ / _ _ / _ _ | _ _ / _ _ / _ _  ☐Continuing |  |
|  |  | ☐ mg  ☐ g  ☐ ml  ☐ mcg  ☐ drops  ☐tsp  ☐tablet/capsule  ☐don’t know  ☐ other:_____ |  | ☐ oral  ☐ topical  ☐ IM  ☐ rectal  ☐ IV  ☐ don’t know  ☐ other : ________ | _ _ / _ _ / _ _ | _ _ / _ _ / _ _  ☐Continuing |  |
|  |  | ☐ mg  ☐ g  ☐ ml  ☐ mcg  ☐ drops  ☐tsp  ☐tablet/capsule  ☐don’t know  ☐ other:_____ |  | ☐ oral  ☐ topical  ☐ IM  ☐ rectal  ☐ IV  ☐ don’t know  ☐ other : ________ | _ _ / _ _ / _ _ | _ _ / _ _ / _ _  ☐Continuing |  |

**Did the patient take any traditional medicines along with PQ at the time of treatment or in the two-week period prior to the onset of the event?**

**☐ Yes ☐No**

*If yes, give details if known:* **______________________________________________________________________________________________________________________________________________________________________________________________________________**

**End of study assessment**

The patient completed follow up until day 16 ☐ Yes ☐No

***If no***

Principal reason for premature termination:

☐Patient vomited the study drug a second time and received rescue treatment before PQ was given

☐Early Treatment failure before PQ was given

☐Patient found to be severely G6PD deficient and excluded before PQ treatment was given

☐Patient withdrew consent

☐Lost to follow up

☐Death

☐ Other: please specify………………………………………………

# Annex X

Signs and symptoms that justify an unscheduled visit:

- Sudden onset of fever
- Disorientation / dizziness / impaired consciousness
- Severe gastro-intestinal symptoms
- Rash
- Severe and persistent headache
- Signs of severe haemolysis
- Signs of severe methemoglobinemia
- Any other sign of illness that worries the patient enough to seek care

# Annex XI

Timeline

| **Month** | **1** | **2** | **3** | **4** | **5** | **6** | **7** | **8** | **9** | **10** |
| --- | --- | --- | --- | --- | --- | --- | --- | --- | --- | --- |
| **Protocol development** | X |  |  |  |  |  |  |  |  |  |
| **Ethics approval** |  | X |  |  |  |  |  |  |  |  |
| **Site preparation** |  | X | X |  |  |  |  |  |  |  |
| **Patient enrolment** |  |  |  | X | X | X | X | X | X |  |
| **Study closure** |  |  |  |  |  |  |  |  | X |  |
| **Analysis and publication** |  |  |  |  |  |  |  |  | X | X |

1. defined as a child whose growth standard is below –3 z-score, has symmetrical oedema involving at least the feet or has a mid-upper arm circumference < 110 mm) [↑](#footnote-ref-1)
